# Mathematical Model of Ammonia Handling in the Rat Renal Medulla

**DOI:** 10.1371/journal.pone.0134477

**Published:** 2015-08-17

**Authors:** Lorette Noiret, Stephen Baigent, Rajiv Jalan, S. Randall Thomas

**Affiliations:** 1 CoMPLEX, University College London (UCL), London, United Kingdom; 2 Mathematics, UCL, London, United Kingdom; 3 Institute of Hepatology, UCL Medical School, London, United Kingdom; 4 IR4M (UMR8081), Université Paris-Sud, Centre National de la Recherche Scientifique, Orsay, France; Emory University, UNITED STATES

## Abstract

The kidney is one of the main organs that produces ammonia and release it into the circulation. Under normal conditions, between 30 and 50% of the ammonia produced in the kidney is excreted in the urine, the rest being absorbed into the systemic circulation via the renal vein. In acidosis and in some pathological conditions, the proportion of urinary excretion can increase to 70% of the ammonia produced in the kidney. Mechanisms regulating the balance between urinary excretion and renal vein release are not fully understood. We developed a mathematical model that reflects current thinking about renal ammonia handling in order to investigate the role of each tubular segment and identify some of the components which might control this balance. The model treats the movements of water, sodium chloride, urea, NH_3_ and ${\text{NH}}_{4}^{+}$, and non-reabsorbable solute in an idealized renal medulla of the rat at steady state. A parameter study was performed to identify the transport parameters and microenvironmental conditions that most affect the rate of urinary ammonia excretion. Our results suggest that urinary ammonia excretion is mainly determined by those parameters that affect ammonia recycling in the loops of Henle. In particular, our results suggest a critical role for interstitial pH in the outer medulla and for luminal pH along the inner medullary collecting ducts.

## Introduction

The kidney is one of the organs that release ammonia into the circulation (unless otherwise specified, ‘ammonia’ refers to both NH_3_ and ${\text{NH}}_{4}^{+}$). Renal ammonia metabolism contributes to acid-base homeostasis [1–5] and is one of the main determinants of plasma ammonia levels (along with the liver); yet the mechanisms controlling renal ammonia handling are not fully understood.

Renal ammonia handling can be decomposed into two main steps: the renal production of ammonia, and the distribution of its subsequent exit between urine and the general circulation. This paper focuses on the mechanisms affecting this distribution between excretion and recovery. Under normal conditions, between 30 and 50% of the ammonia produced in the kidney is excreted in the urine. This fraction can change drastically under pathological conditions. In particular, it is modified under conditions that alter plasma pH, ammonia concentrations, or potassium concentrations [3, 6, 6–8]. For instance, in acidosis, 75% of newly formed ammonia is excreted in the urine, whereas in alkalosis the fraction excreted drops to 20% [3].

Within the renal medulla, pH and potassium concentrations are known to affect ammonia transport, since in vitro microperfusion studies showed that ${\text{NH}}_{4}^{+}$ and NH_3_ transport are influenced by pH and potassium concentration in the microenvironment (e.g., [9–14]). Yet, predicting how a pathological change (e.g., in plasma pH, or ammonia) may impact the medullary environment and ammonia transport is difficult, and the mechanisms controlling the balance between urinary and renal vein release remain poorly understood. There are two main reasons for this. Firstly, renal organization is complex (organized along corticomedullary axes) and involves numerous components. As a result, predicting how a change in one transporter or solute concentration will impact the overall dynamics is extremely difficult without a theoretical formalism. Secondly, uncertainties remain regarding the renal physiological microenvironment. Micropuncture studies have allowed direct measurements of physiological conditions, but only in accessible cortical and papillary (inner medulla) regions, providing a partial view of cortical and juxtamedullary nephrons [10–12, 15, 16]. Changes in pH and potassium concentration gradients within the medulla resulting from pathological conditions are unknown.

In this study, we use a mathematical model of medullary ammonia transport in the rat to help understand the contributions of the various tubular segments to urinary ammonia excretion. The model helps us to identify the medullary physiological factors that might be associated with an increase or decrease in ammonia excretion. In particular, we are interested in predicting the impact of an intervention (such as inhibition of a transporter or changes in pH concentrations) on the percentage of ammonia excreted in urine (fractional excretion). The simulation results reported here suggest that urinary ammonia is notably controlled by parameters that favor luminal secretion into the descending limb of the loops of Henle resulting in a recycling effect and NH_3_ secretion into collecting ducts.

## Results

### Model overview

**Fig 1:**
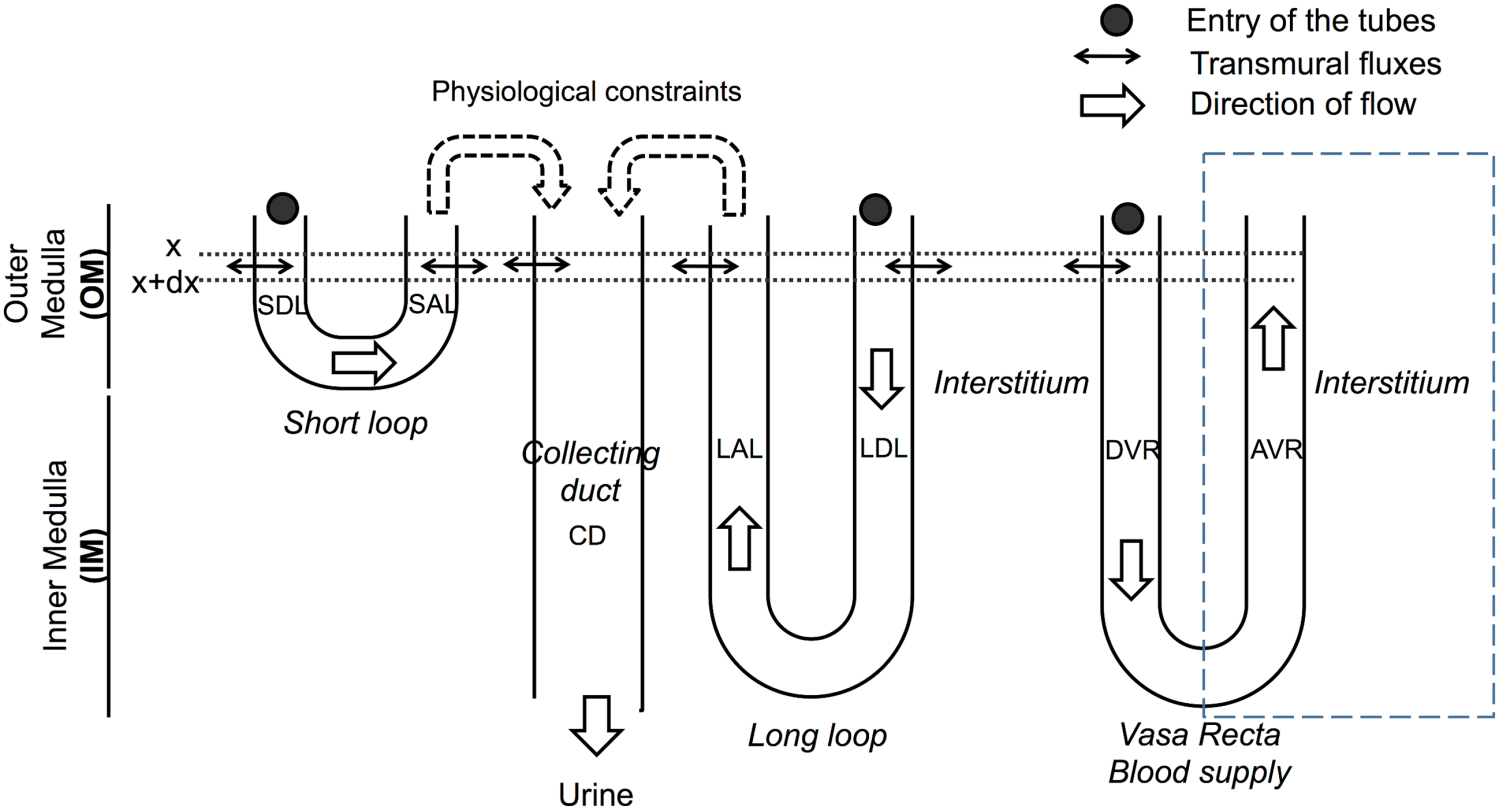
Schematic diagram of the model: medullary structures. The model distinguishes short and long nephrons. The flows are set at the entry to the descending limbs and vasa recta (solid circles). Flows are then calculated along each tube using the equations for transmural fluxes. Inflow to the collecting ducts (CD) is calculated from the flows leaving the ascending limbs and under some physiological constraints (see [17] and text). The ascending vasa recta (AVR) is lumped with the interstitium. The figure does not depict the virtual shunts within the inner medulla that connect the descending and ascending part of the loops of Henle and vasa recta; these shunts are used to replicate the experimentally observed decrease in the number of tubes within the inner medulla. SDL: short descending limb, SAL: short ascending limb, LAL: long ascending limb (includes the thin ascending limb in the IM and the thick ascending limb in the OM), LDL: long descending limb, DVR: descending vasa recta.

The model illustrated in Fig 1 represents the transport of water and solutes (NaCl, urea, NH_3_, ${\text{NH}}_{4}^{+}$, and an unspecified non-reabsorbable solute (NRS) which includes the effect of KCl in the collecting ducts) in an idealized rat renal medulla. The model equations describe the variations in volume and solutes flows resulting from electrochemical gradients and active transport. Differences of electrical potential, pH, and potassium concentration gradients are imposed at each depth; this allows us to evaluate the role of these three factors in ammonia handling (see the parameter studies below) while keeping the size and complexity of the model manageable. These gradients directly affect NH_3_ and ${\text{NH}}_{4}^{+}$ transmural fluxes. Model outputs of each simulation scenario give the concentration and flow profiles along each medullary structure. Most of the parameter values were taken from the rat literature. To identify the parameters associated with a change in urinary ammonia excretion, a partial sensitivity analysis was performed; starting from our baseline (control) scenario, each parameter value (e.g., NH_3_ permeability in the outer stripe collecting duct) was perturbed and the changes in renal ammonia transport were analyzed.

### Baseline scenario

#### Simulation results compared to micropuncture literature results

**Fig 2:**
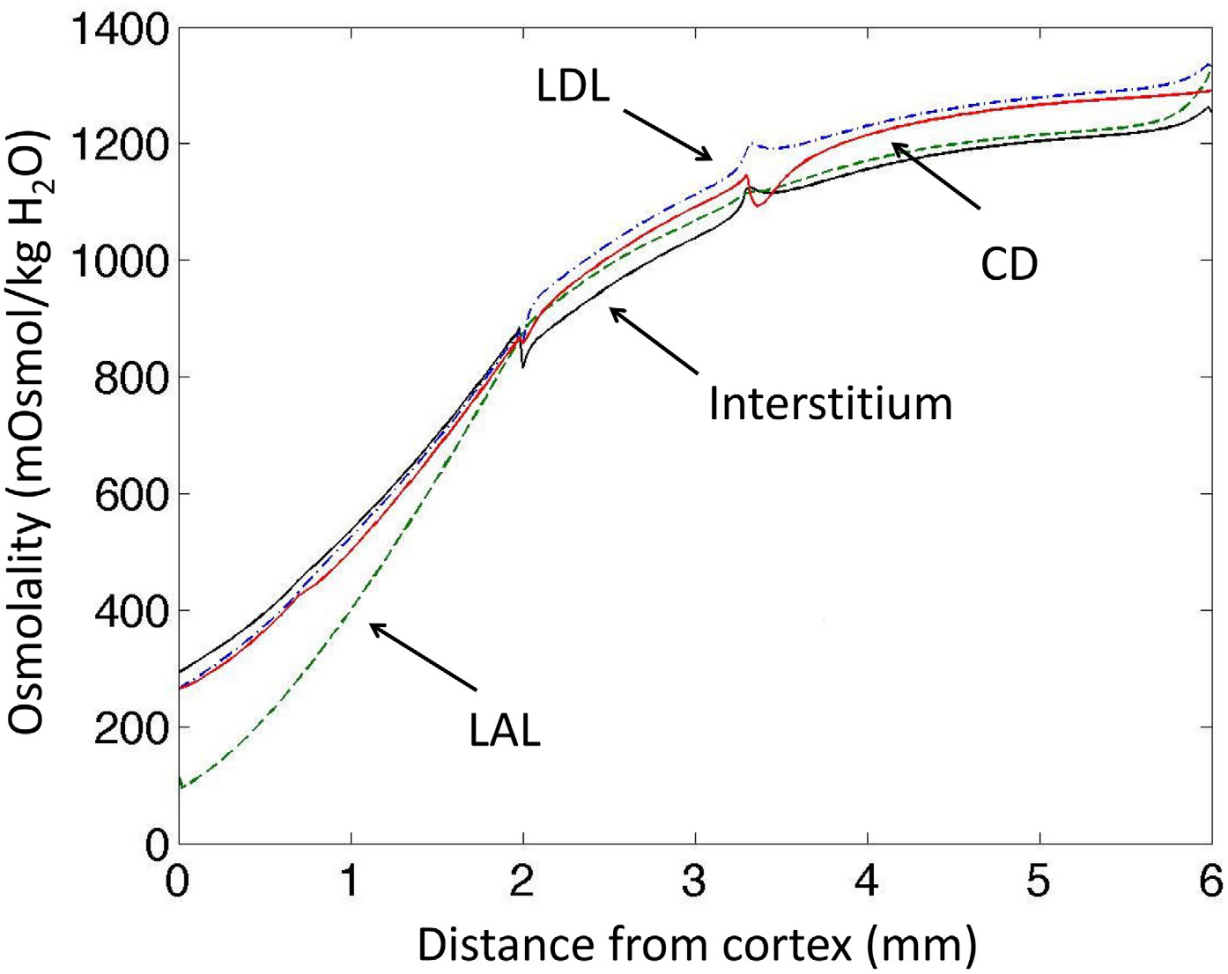
Model results: osmolality gradients (mOsm/KgH_2_O). The osmolality increases in the inner medulla thanks to our introduction of interstitial external osmoles as a surrogate concentration mechanism in the inner medulla. In our model, transport parameters are defined for each region (OS, IS, UIM and LIM), and therefore small discontinuities can be observed around the junctions of the regions.

**Fig 3:**
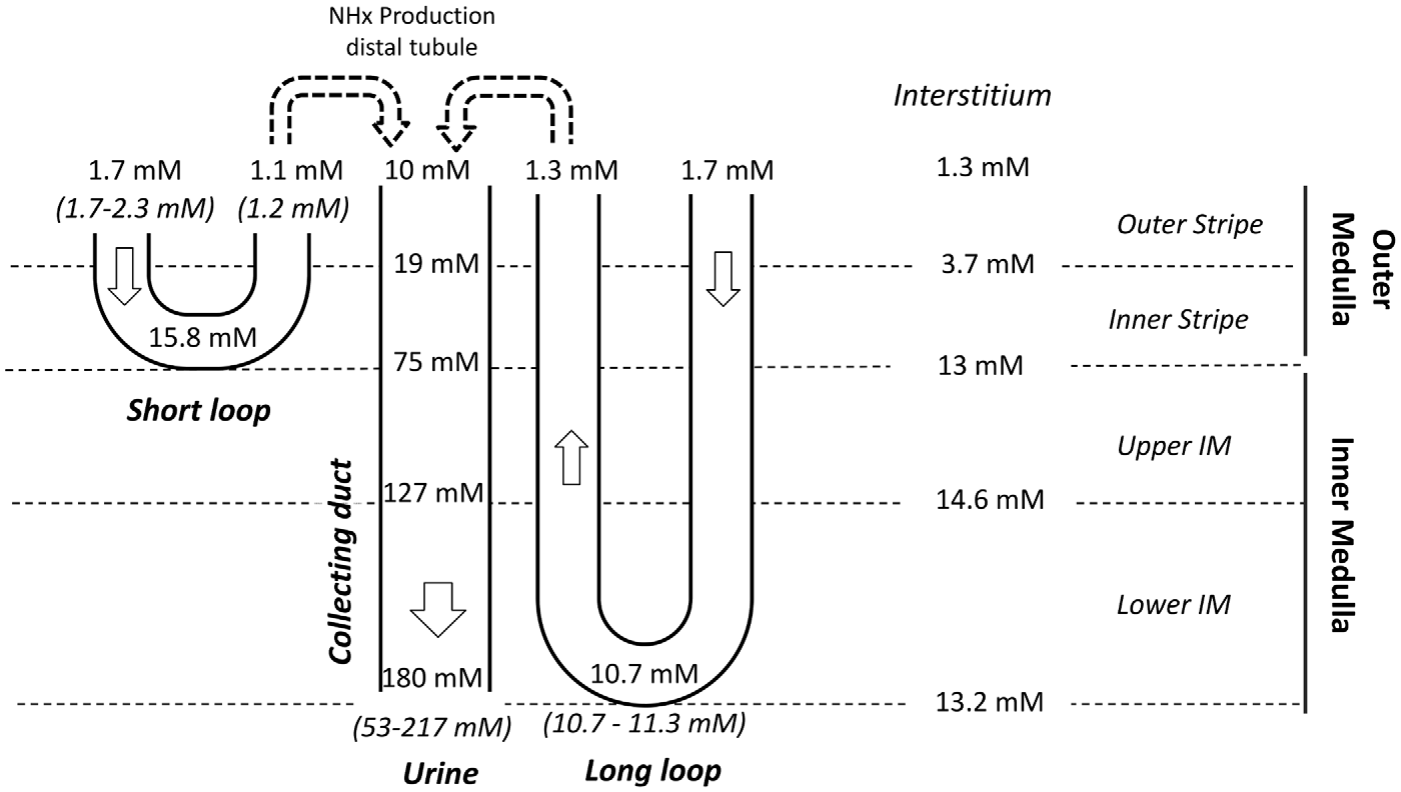
Simulated total ammonia concentrations compared to micropuncture measurements *(values in italics)* [10, 11, 18–20]. The baseline scenario is consistent with experimental measurements.

**Fig 4:**
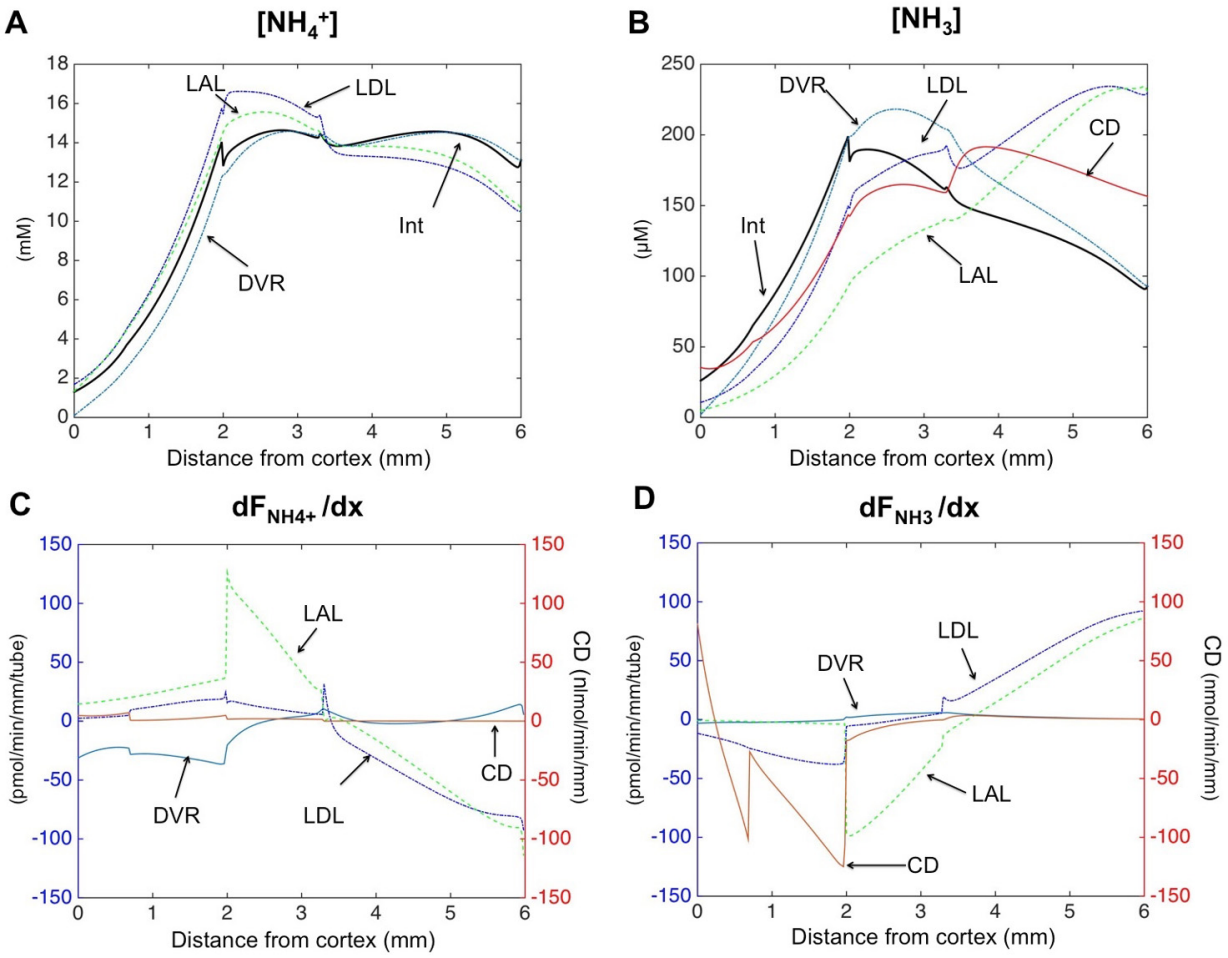
Model results: (A-B) ${\text{NH}}_{4}^{+}$ and NH_3_ concentration profiles. In the outer medulla, passive diffusion gradients favor secretion of NH_3_ into nephron segments and reabsorption of ${\text{NH}}_{4}^{+}$. (C-D) ${\text{NH}}_{4}^{+}$ and NH_3_ transmural fluxes profiles. Positive fluxes denote absorption, whereas negative fluxes represent secretion. Please note the different scales for total fluxes in the collecting ducts (nmol.min^-1^mm^-1^), whereas fluxes are given per tube in nephron segments and blood vessels (pmol.min^-1^mm^-1^.tube^-1^).

**Fig 5:**
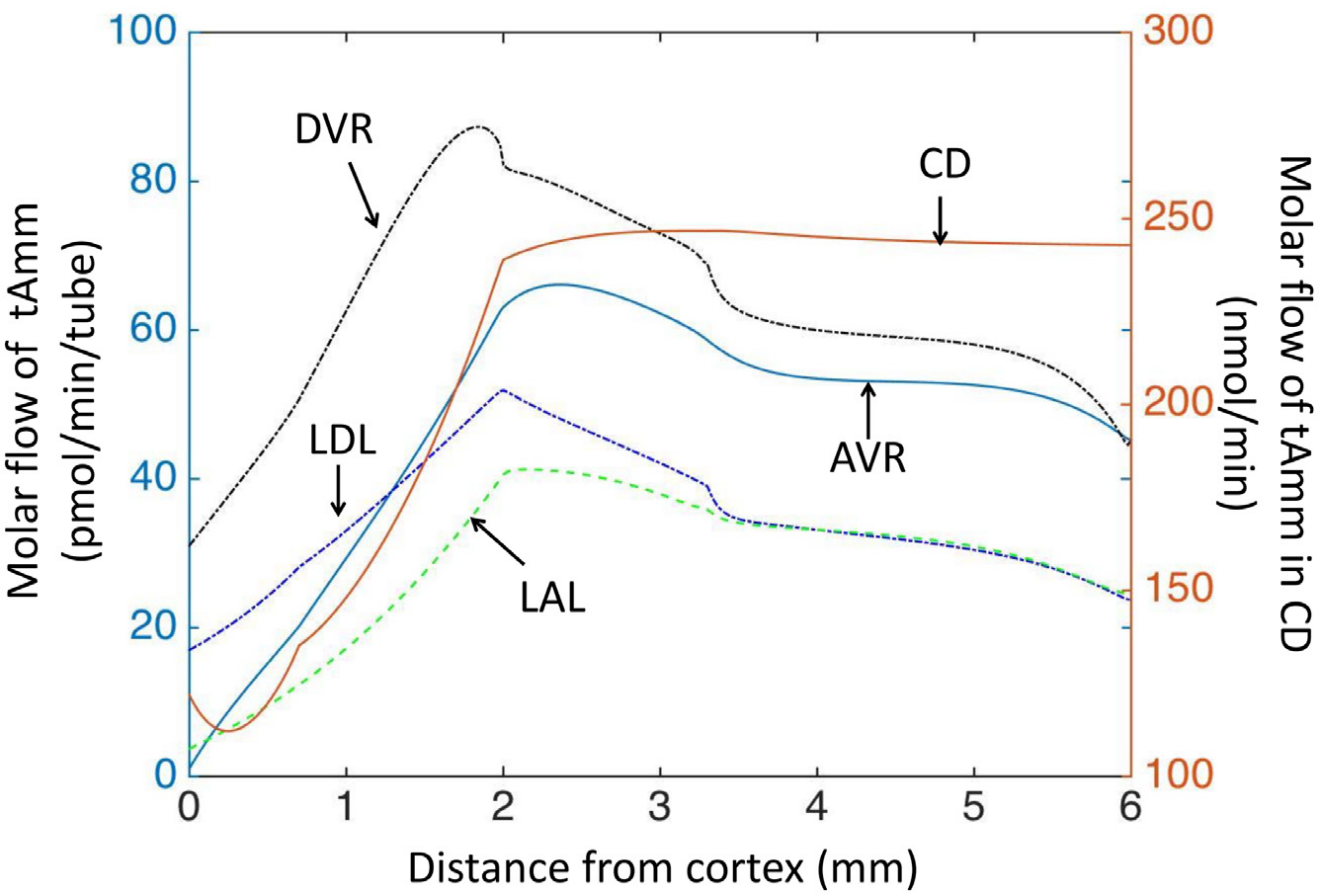
Model results: flow of total ammonia (tAmm) in each structure under baseline conditions. Ammonia is reabsorbed in OM ascending limb of the loops of Henle (decreased flow), and partly recycled into descending limbs (increased flow) or secreted into the collecting ducts. The increase in ammonia flow in the OM descending limbs is also due to tubular ammonia production. Please note the different scale for total flow in the collecting ducts, whereas flows are given per tube in nephron segments and blood vessels.

The baseline scenario reproduces experimental data regarding osmolality in antidiuresis (Fig 2), and the model outputs are similar to the profiles presented in Hervy et Thomas [17]. The model’s baseline ammonia concentration profiles are close to experimental measurements obtained by micropuncture (Figs 3 and 4). Predicted ammonia concentration is 10.7 mM at the papillary tip of the loops of Henle (versus 10.7–11.3 mM measured by Buerkert et al., [10, 18]), 1.1 mM at the exit of short nephrons (versus 1.2 mM measured in early distal tubule in [11]), and 180 mM in urine (measured urinary concentration varies between 53 mM and more than 200 mM, [19, 20]). The percentage of ammonia flow reaching the papillary tip of the nephron ($\%tip=\frac{F^{LDL}(L)}{F^{LDL}(0)}$) represents 140% of end proximal delivery (Fig 5). This accumulation of ammonia is mainly due to ammonia production along the descending limbs (in simulations without medullary production, ammonia concentration at the papillary tip only reached 8 mM instead of 10.7 mM and ammonia flow (per nephron) at the tip is similar to LDL inflow). In the medullary thick ascending limbs (MTAL), ${\text{NH}}_{4}^{+}$ was reabsorbed (as reported experimentally), and the flow out of MTAL into the distal tubules is ∼ 20% of the delivery to the loops of Henle (constraint imposed on ${\text{Vmax}}_{NH_{4}^{+}}^{AL}$ value). In the model, 72% of the reabsorption in MTAL is mediated by short nephrons. We calculated the fraction of ${\text{NH}}_{4}^{+}$ transported via active transport at each point along the length thick ascending limbs. 67% of ${\text{NH}}_{4}^{+}$ reabsorption in MTAL is carrier mediated, which is consistent with in vitro experiments (64% in [21]). Ammonia is secreted into the outer medullary collecting ducts (OMCD), but not into the inner medullary collecting ducts (IMCD). Predicted secretion in collecting ducts accounts for 50% of urinary ammonia content, which is comparable to experimental measurements. Baseline urinary ammonia excretion represents 60% of the amount of ammonia delivered at the entry to the descending limbs.

#### Magnitude of NH_3_ transmural fluxes

For pH in the physiological range, NH_3_ concentrations are at least 100 times lower than ${\text{NH}}_{4}^{+}$ concentrations (pKa = 9.05). Hence, total ammonia concentrations tAmm are essentially equal to ${\text{NH}}_{4}^{+}$ concentrations. However, even though NH_3_ concentrations are low compared to ${\text{NH}}_{4}^{+}$ levels, transmural fluxes of NH_3_ are quantitatively important, because NH_3_ permeability is two orders of magnitude higher than ${\text{NH}}_{4}^{+}$ permeability. Concentration gradients favor NH_3_ secretion in the outer medulla (into nephrons, collecting ducts, and vasa recta), whereas electro-diffusional transport for ${\text{NH}}_{4}^{+}$ is essentially in the opposite direction (reabsorption) (Fig 4). According to the model predictions, transmural fluxes of NH_3_ are mainly responsible for overall ammonia secretion in outer medullary descending limbs and in collecting ducts. In particular in the model, NH_3_ secretion systematically dominates ${\text{NH}}_{4}^{+}$ transmural fluxes in the collecting ducts. In thick ascending limbs, on the other hand, ${\text{NH}}_{4}^{+}$ transport dominates NH_3_ transport (results not shown).

#### Ammonia recycling in the loops of Henle

${\text{NH}}_{4}^{+}$ absorbed from the thick ascending limbs can be secreted directly into the collecting ducts for excretion, or it can be recycled into the descending limbs and the descending vasa recta thereby promoting medullary accumulation. NH_3_ secretion in DL results in higher total ammonia flows and concentrations at the entry of the MTAL, which in turn favors ammonia reabsorption through ${\text{NH}}_{4}^{+}$ active transport. The process of ${\text{NH}}_{4}^{+}$ reabsorption from the MTAL followed by NH_3_ secretion into the DL allows ultimately a larger fraction of ammonia to be secreted into collecting ducts (see next section).

### Effects of local perturbations

This section reports the results of the parameter analysis, whose aim was to identify the model parameters that most influence the rate of urinary ammonia excretion.

#### Sensitivity to transport parameters

**Fig 6:**
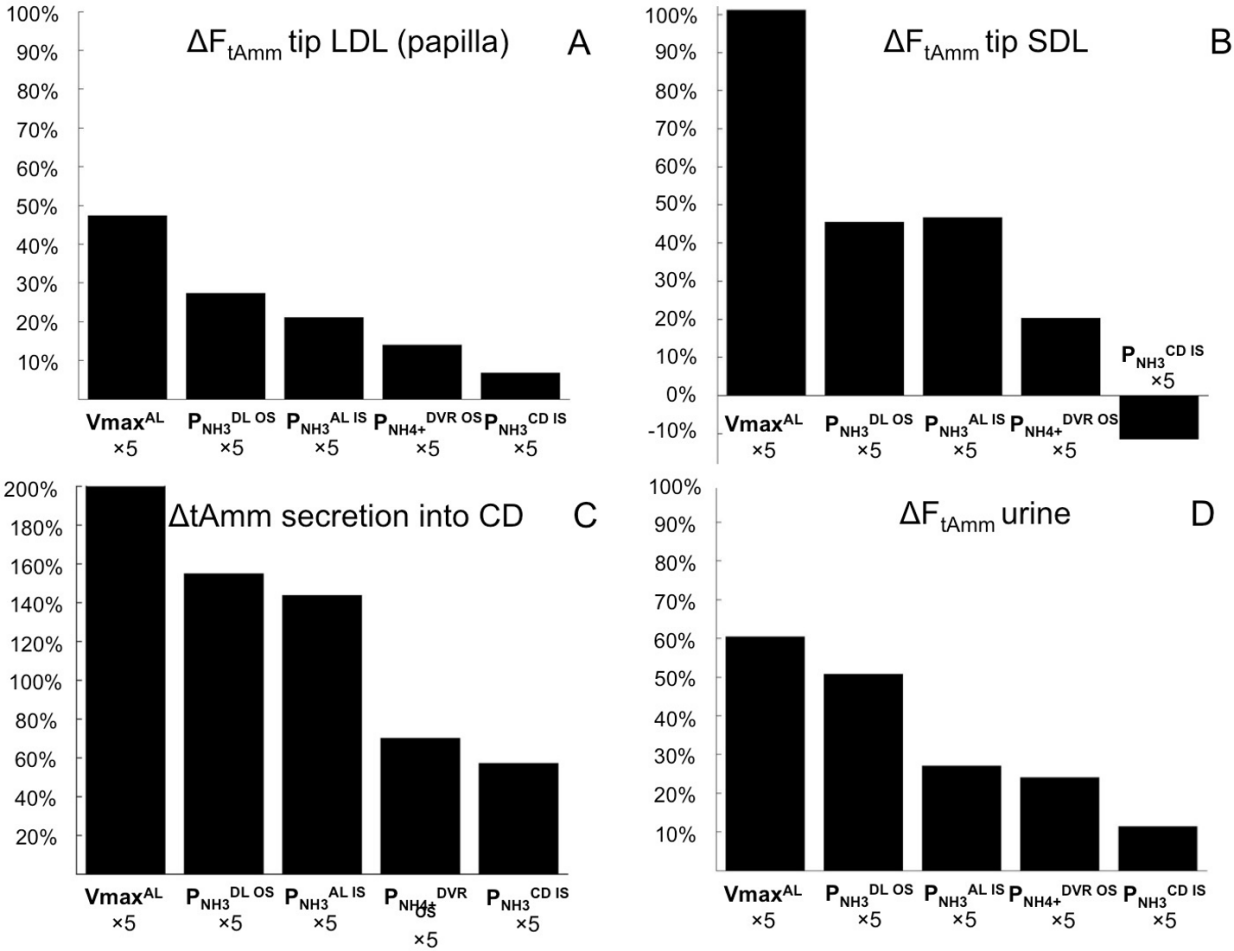
Parameter study. %changes in total ammonia flows resulting from multiplying each baseline parameter by 5: A) changes at the papillary tip of long loops, B) changes at the tip of short loops (outer-inner medullary junction), C) changes in total ammonia secretion into the collecting ducts, D) changes in urinary excretion. Parameters associated with ammonia recycling in the loops of Henle (especially of short nephrons, see B) are associated with the largest increase in urinary ammonia flow (D). The figure only shows the parameters that affect urinary ammonia excretion by at least 10%. Vmax^*AL*^: maximum rate of active transport of ${\text{NH}}_{4}^{+}$ in thick ascending limbs; ${\text{P}}_{NH_{3}}^{DLOS}$ / ${\text{P}}_{NH_{3}}^{ALIS}$ / ${\text{P}}_{NH_{3}}^{CSIS}$ NH_3_ permeability of outer stripe descending limbs/ inner stripe ascending limbs/ inner stripe collecting ducts; ${\text{P}}_{NH_{4}^{+}}^{DVRIS}$ ${\text{NH}}_{4}^{+}$ permeability in descending vasa recta of the inner stripe.

To simulate the impact on ammonia excretion of a change in membrane properties, each transport parameter (NH_3_ and ${\text{NH}}_{4}^{+}$ permeabilities and Vmax) was successively multiplied by a factor *k* (*k* ranging from 0 to 100). Results obtained when one parameter at a time was perturbed by a factor k = 5 are presented in Fig 6. In general, parameters favoring ammonia (NH_3_ or ${\text{NH}}_{4}^{+}$) secretion into the (short) loops of Henle, such as Vmax for ${\text{NH}}_{4}^{+}$ in the MTAL or NH_3_ permeability in the outer medullary descending limbs, increase the rate of urinary ammonia excretion. This is because ammonia recycling in the loops of Henle leads to increased delivery of ammonia to the thick ascending limbs, thereby favoring ammonia reabsorption from the thick ascending limbs and ultimately secretion into the collecting ducts. Specifically these parameters are:

- ${\text{Vmax}}_{{\text{NH}}_{4}^{+}}^{\text{AL}}$: reabsorption of ${\text{NH}}_{4}^{+}$ from the thick ascending limbs has a cumulative effect on urinary excretion. First, it increases the direct delivery of ammonia to the collecting ducts, and second, it favors ammonia recycling in the loops of Henle.
- ${\text{P}}_{{\text{NH}}_{3}}^{\text{DL OS}}$: this parameter controls NH_3_ recycling in the loops of Henle. Given that the concentration gradient of NH_3_ across the walls of the descending limbs in the outer stripe is inwards, an increase in ${\text{P}}_{{\text{NH}}_{3}}^{\text{DL OS}}$ favors NH_3_ diffusion into long and short descending limbs, contributing to a higher flow of total ammonia (tAmm) in short nephrons. As a result, a larger amount of ${\text{NH}}_{4}^{+}$ is delivered and thus reabsorbed from the short MTAL. NH_3_ permeability in the inner stripe of the descending limbs, ${\text{P}}_{{\text{NH}}_{3}}^{\text{DL IS}}$, also has a positive effect on urinary flow, but it is less potent than ${\text{P}}_{{\text{NH}}_{3}}^{\text{DL OS}}$.
- ${\text{P}}_{{\text{NH}}_{4}^{+}}^{\text{AL IS}}$: the lumen positive voltage favors ${\text{NH}}_{4}^{+}$ reabsorption from the MTAL, hence an increase in ${\text{NH}}_{4}^{+}$ permeability is associated with higher ammonia excretion.
- ${\text{P}}_{{\text{NH}}_{4}^{+}}^{\text{DVR OS}}$: in the baseline scenario, NH_3_ and ${\text{NH}}_{4}^{+}$ concentration gradients favor secretion into the DVR OS. Hence, an increase in ${\text{NH}}_{4}^{+}$ permeability, ${\text{P}}_{{\text{NH}}_{4}^{+}}^{\text{DVR OS}}$, leads to higher secretion in the DVR OS. This results in higher ammonia flows and concentrations downstream in the inner medullary descending and ascending vasa recta, and hence in the interstitium since the AVR is lumped with the interstitium in our model. The increase in ammonia concentration in the interstitium limits the reabsorption of ammonia from the nephrons and promotes its secretion into the collecting ducts.
- ${\text{P}}_{{\text{NH}}_{3}}^{\text{CD IS}}$: an increase in NH_3_ permeability in the collecting ducts inner stripe leads to a mild effect on urinary excretion by favoring NH_3_ secretion into the collecting ducts.

#### Inhibition of the recycling effect (${\text{P}}_{{\text{NH}}_{3}}^{\text{DL OM}}=0$)

**Fig 7:**
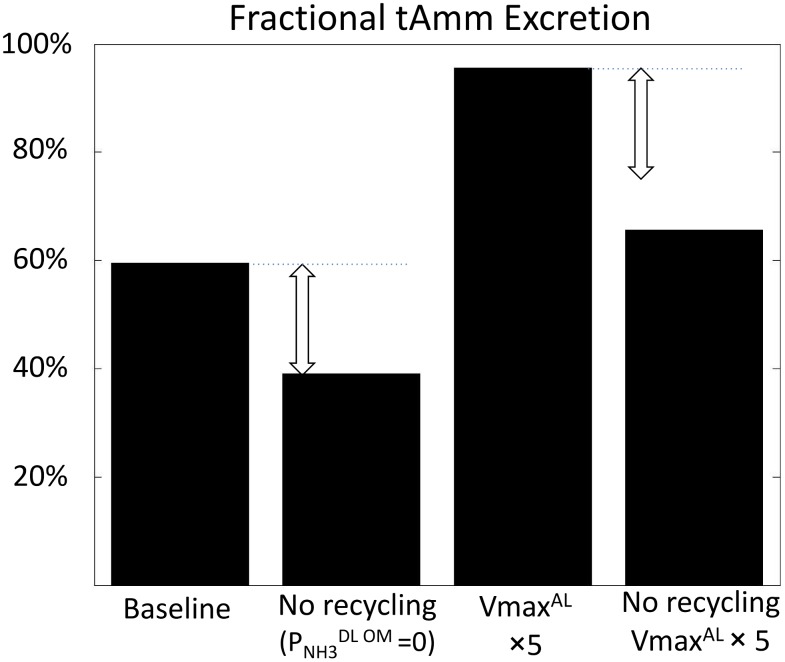
Inhibition of NH_3_ secretion in the DL OM (permeability ${\text{P}}_{{\text{NH}}_{3}}^{\text{DL OM}}=0$) prevents ammonia recycling in the loops of Henle, which limits urinary ammonia excretion. The effect is more potent when ammonia reabsorption in the MTAL is increased (maximum rate of active transport ${\text{Vmax}}_{{\text{NH}}_{4}^{+}}^{\text{AL}}\times 5$).

The parameter analysis suggested that the parameters promoting NH_3_ recycling in the loops of Henle also promote high urinary ammonia excretion. To test the importance of the recycling effect, NH_3_ permeability in the outer medullary descending limbs (OS and IS) was set to 0 (Fig 7). As expected, this resulted in a lower ammonia flow at the bend of the loops of Henle (-49% in short nephrons, -26% at the papillary tip of long nephrons). Inhibition of NH_3_ DL permeability was also associated with a 34% decrease in the absolute urinary ammonia flow, which demonstrates the importance of the recycling effect. Ammonia recycling also amplifies the effect of other parameter changes. For instance, the increase in urinary ammonia excretion observed when the active transport is increased in the MTAL (${\text{Vmax}}_{{\text{NH}}_{4}^{+}}^{\text{AL}}\times 5$) is lower when the recycling in the loops of Henle is prevented (Fig 7).

#### Inhibition of NH_3_ secretion in collecting ducts (simulation of Rh C glycoprotein deletion)

In the baseline scenario, NH_3_ secretion into collecting ducts contributes significantly to urinary ammonia excretion. We therefore simulated a scenario in which NH_3_ permeabilities along the collecting ducts were decreased by 67%; this corresponds to the decrease in permeability reported in Rhcg knock-out mice [22]. Lowering NH_3_ permeability in collecting ducts decreases ammonia excretion rate by 15%; this result is lower but still comparable to the decrease in ammonia excretion reported in mice with Rh C glycoprotein deletion (-27% in [23]).

#### Increased active transport in collecting ducts (${\text{Vmax}}_{NH_{4}^{+}}^{CD}$)

The inner medulla is often considered an important site for ammonia secretion ([19, 24, 25], review [26]). However, in our baseline scenario, ammonia secretion in the IMCD is negligible. One reason could be that the maximum rate of active transport selected in baseline (${\text{Vmax}}_{NH_{4}^{+}}^{CD}$) is too low. We investigated a scenario with a higher rate (${\text{Vmax}}_{NH_{4}^{+}}^{CD}$ multiplied by 100); urinary ammonia increased by 29% due to an increased secretion in the upper IMCD. This secretion resulted in a low ammonia concentration and fractional delivery at the papillary tip of the loops of Henle (concentration: 5.3 mM vs 10.7 mM in baseline, fractional delivery: 70% vs 140% in baseline).

#### Sensitivity to the concentration of external osmolytes

The external osmoles, *E*, introduced in the inner medullary interstitium help to concentrate tubular fluid and urine. As shown in Table 1, this parameter greatly impacts urinary ammonia concentration, but not the fractional excretion.

**Table 1:** Effects of electrical potential ΔV in the whole medulla and inner medullary external osmoles E^IM^ on ammonia excretion.

	k	ΔTip SDL / LDL	Δ Top SAL / LAL	ΔUrine
ΔV All segments	0.5	-9% / -6%	17% / 19%	-7%
	2	19% / 11%	-27% / -30%	14%
ΔV AL	0.5	-10% / -6%	17% / 19%	-6%
	2	20% / 12%	-27% / -30%	13%
ΔV CD	0.5	0% / 0%	0% / 0%	-1%
	2	-1% / -1%	-1% / -1%	1%
E^IM^	0.5	-5% / 2%	2%/ 37%	3%
	2	8% / 4%	1% / -43%	-5%

Baseline value for each parameter is halved (*k* = -0.5) or doubled (*k* = 2). The results are presented as the percentage changes from baseline. The electrical potential has a small effect on urinary ammonia excretion.

#### Sensitivity to the transmembrane electrical potential

Transmembrane voltages ΔV favor ${\text{NH}}_{4}^{+}$ reabsorption in the MTAL (lumen positive voltage). As shown in the baseline scenario, the majority of ${\text{NH}}_{4}^{+}$ reabsorption in MTAL is carrier mediated, and less than a third of ${\text{NH}}_{4}^{+}$ transmural flux is driven by the lumen positive voltage. A two-fold increase (or decrease) in the transmembrane voltages in the MTAL has only a small impact (inferior to 13%) on urinary ammonia excretion (see Table 1). In the collecting ducts, ${\text{NH}}_{4}^{+}$ transmural fluxes are negligible compared to NH_3_, and thus a change in the potential does not affect the urinary ammonia excretion.

#### Sensitivity to a change in pH

To test the influence of pH, the pH gradient was modified by changing pH by plus/minus 0.2 pH units at the entry or exit of each tube in turn. Then a wider range of pH variation is used to explore the role of urinary pH. In the model, the pH at each depth is interpolated linearly between the entry and exit of each tube; therefore, changes at the entry of the tube mainly affect the pH throughout the outer medulla, whereas a change at the papilla level affects mainly the pH throughout the inner medulla. The results are summarized in Table 2.

**Table 2:** Effects of pH changes.

	Baseline pH − 0.2			Baseline pH + 0.2		
	ΔTip SDL/LDL	ΔOutlet SAL/LAL	ΔUrine	ΔTip SDL-LDL	ΔOutlet SAL-LAL	ΔUrine
**Int cortico-med.**	-22% / -25%	-10% / -10%	**-37%**	21% / 21%	9% / 8%	**46%**
Int papilla	0% / 8%	2% / 3%	-7%	-1% / -10%	-2% / -3%	7%
DL inlet	18% / 12%	7% / 4%	8%	-21% / -13%	-8% / -5%	-12%
DL-AL bend loop	4% / 23%	1% / 1%	0%	-5% / -22%	-1% / -1%	0%
AL outlet	0% / -4%	1% / 2%	-1%	0% / 4%	-1% / -3%	1%
CD inlet	-4% / 9%	-4% / -4%	15%	2% / -9%	3% / 2%	-14%
CD outlet	-9% / -30%	-6% / -6%	9%	11% / 41%	8% / 8%	-10%
DVR inlet	1% / 1%	0% / 0%	1%	-1% / -1%	0% / 0%	-1%

pH is modified (baseline pH ± 0.2) at one end of the tube (cortico-medullary junction or papilla), the other values are deduced by linear interpolation. The results are presented as the percentage changes from baseline. Urinary ammonia excretion is most strongly affected by a change in the interstitial pH (bold underlined text).

**Fig 8:**
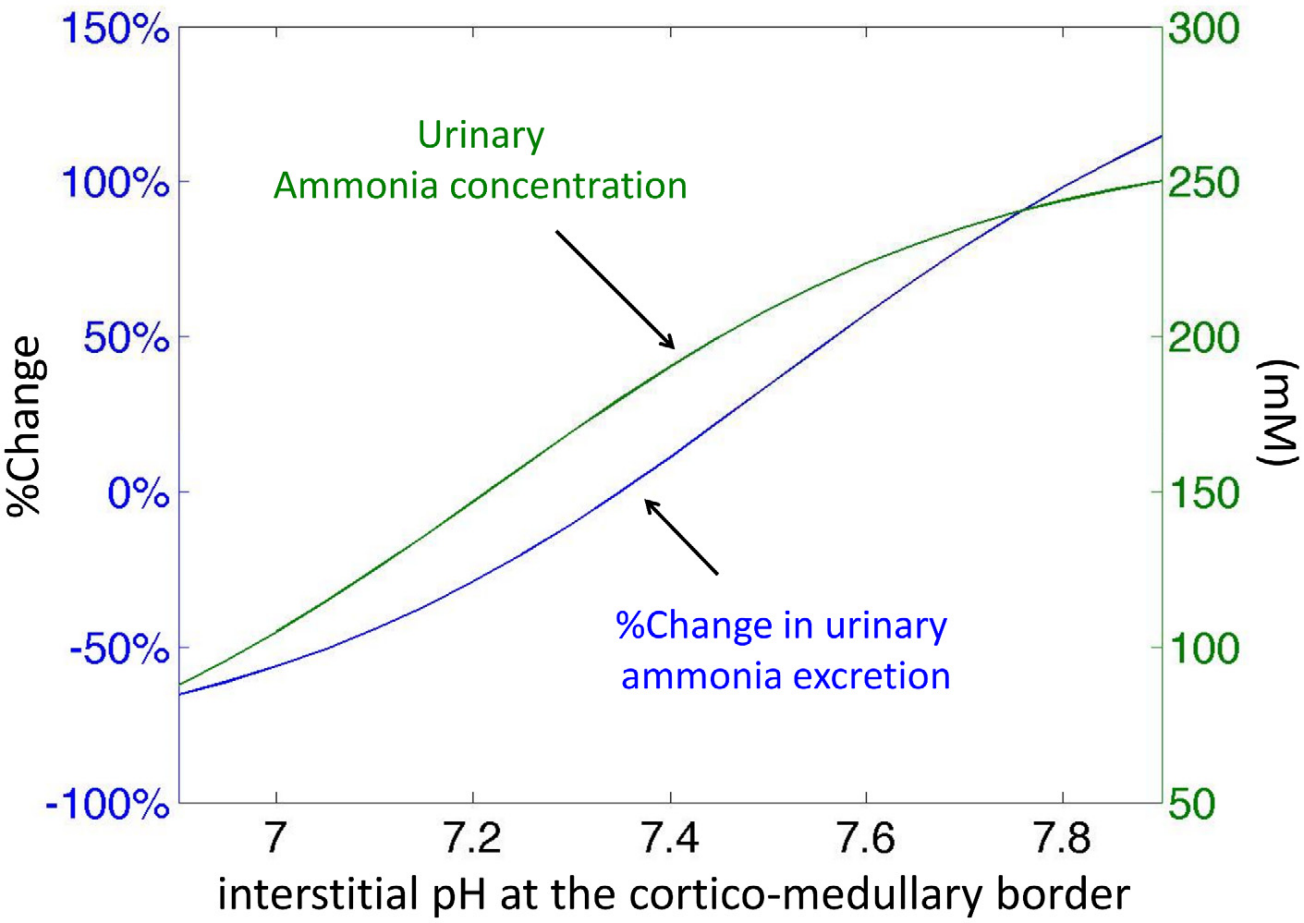
Impact of pH environment on urinary excretion of ammonia. The figure shows the percentage change in ammonia excretion rate and the urinary total ammonia concentration when the pH at the top of the interstitium is varied (thus changing the medullary pH profile).

Urinary ammonia excretion is very sensitive to a change in pH at the top of the interstitium, which reflects the importance of the pH environment of the outer medulla (Fig 8). In particular, alkalinization of the OM interstitium leads to an increase in ammonia excretion rate. The mechanism for this increase is as follows. Alkalinization of the OM interstitium increases NH_3_ concentration in the interstitium and thus favors NH_3_ secretion into the descending limbs and collecting ducts. As described for the transport parameters, a high ammonia flow in the loops of Henle increases the reabsorption in the ascending limbs and also increases secretion in collecting ducts.

Changes in the pH at the entry of the descending limbs or of the collecting ducts impact the ammonia excretion rate, but these effects are smaller than those due to a change in interstitial pH. The mechanism is similar, namely, luminal acidification of one of these segments favors NH_3_ secretion into the lumen of the tube.

We also investigated whether simultaneous acidification of the descending limbs and collecting ducts (-0.2 pH at the top of each tube) produces the same increase in ammonia urinary flow as alkalinization of the interstitium. A combined acidification of the descending limbs and collecting ducts leads to a 23% increase in ammonia excretion rate, which is less than what we obtained with alkalinization of the interstitium (46%).

**Fig 9:**
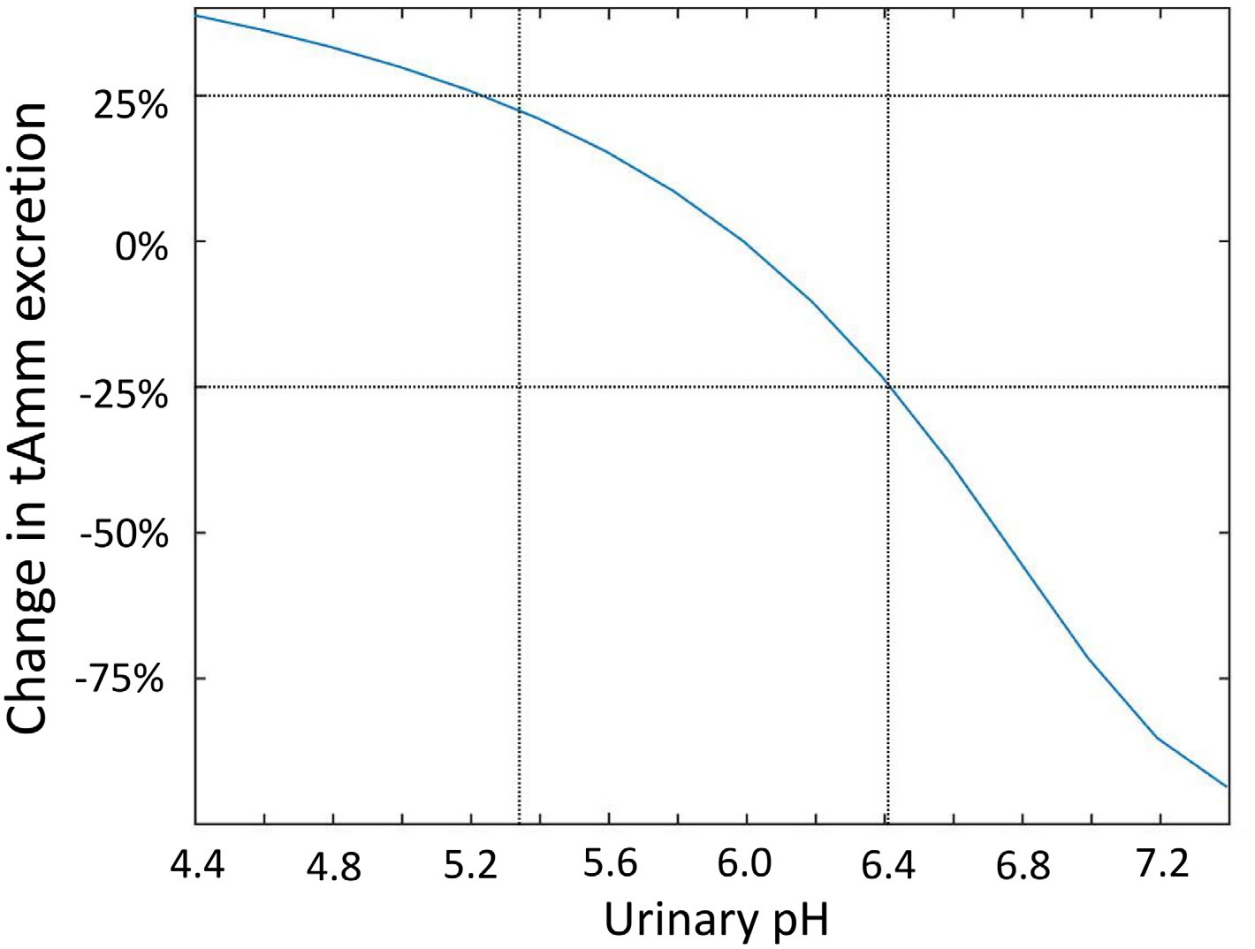
Impact of urinary pH on urinary excretion of ammonia. The figure shows the percentage change in ammonia excretion rate when the pH at the exit of the collecting ducts is varied.

Urinary pH in rats varies from 4.4 to more than 7.4 [27–29]. However, within any given experiment (e.g. alkalosis, acidosis, hyperkalemia), the range of variation is often smaller: the changes from control values could vary from -0.65 to 0.42 [10, 12, 16, 18, 27, 30, 31]; in our model this corresponds to a pH varying from 5.34 to 6.41. Fig 9 shows the impact of varying the pH at the exit of the collecting ducts on urinary ammonia excretion. In the model, NH_3_ transmural fluxes in the collecting ducts and urinary ammonia excretion are strongly related to pH at the exit of the collecting ducts. A more alkaline urinary pH leads to lower NH_3_ secretion in the collecting ducts and hence to lower ammonia excretion. When urinary pH is increased to 6.59 or above, NH_3_ starts to be reabsorbed from the collecting ducts, instead of being secreted into them. However, this should not occur with moderate variations in pH (pH varied from 5.34 to 6.41). Within this range of variation, the changes in ammonia excretion can reach 25%.

#### Changes in interstitial pH and inhibition of NH_3_ secretion in the collecting ducts

When the collecting ducts are impermeable to NH_3_, the increase in urinary flow resulting from an alkalinization of the interstitium OM is not as great as the increase observed with normal permeabilities (change in urinary excretion after alkalinization 54 nmol/min versus 111 nmol/min in baseline).

#### Sensitivity to potassium gradients

Changes in plasma potassium concentrations are known to affect ammonia production and reabsorption in the thick ascending limb. Using in vitro microperfusion of thick ascending limbs, Good compared ammonia reabsorption in moderate (4mM) versus high (24mM) luminal and bath concentrations of potassium [21]. In his study, ${\text{NH}}_{4}^{+}$ reabsorption in the MTAL was halved with high luminal potassium concentration due to decreased active ammonia transport. In our model, high luminal potassium concentrations in the MTAL (potassium concentration set at 24mM everywhere) produced a similar decrease in total ammonia reabsorption in the MTAL (tAmm reabsorption -49% versus -48% in [21]), mainly through a decrease in carrier mediated transport. This resulted in a decrease in urinary excretion (-26%), the lower reabsorption being partly compensated by a higher delivery at the entry of collecting ducts.

To further understand the influence of potassium, the potassium concentrations at the papillary tip of the loops colourredof Henle and interstitium were set to 6 mM (scenario close to Wall and Kroger [25]). This led to greatly increased carrier-mediated ${\text{NH}}_{4}^{+}$ flux. In particular, ammonia fluxes due to active transport in the lower inner medulla increased. The ammonia excretion rate was increased by 18%.

### Sensitivity analysis based on alternative baseline scenarios

We carried out new parameter analyses based on four alternative baseline scenarios.

**Fig 10:**
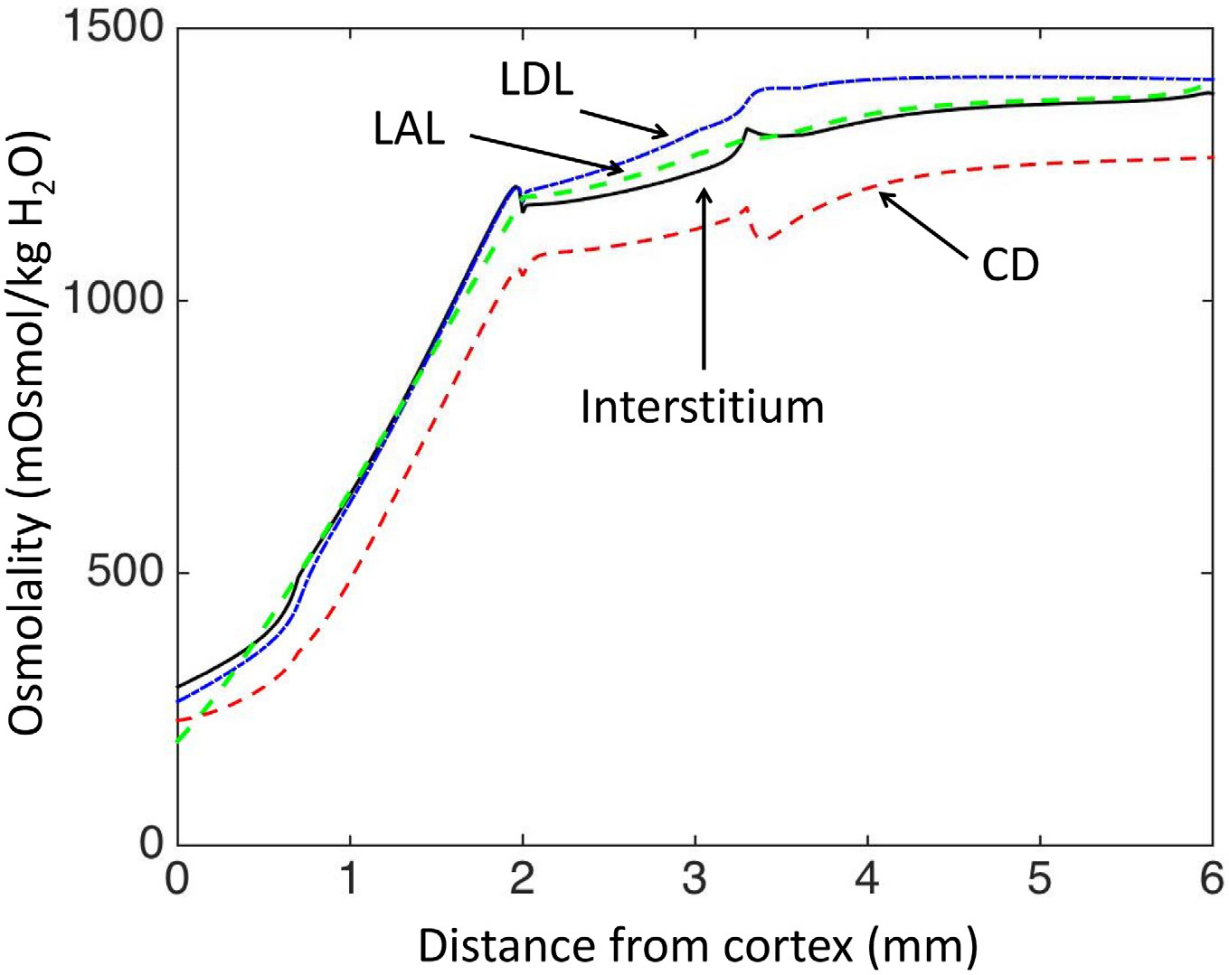
Model results (alternative baseline scenario): osmolality gradients (mOsm/KgH_2_O) obtained when the segments of the descending limbs are assumed to be water impermeable.

In the first alternative scenario, some sub-segments of the descending limbs are assumed to be water impermeable, to reflect the data of Pannabecker and coworkers on aquaporin expression [32, 33]: the outer-stripe of short nephrons, the inner medullary segment of long nephrons whose loops of Henle bend within the first millimeter of the inner medulla, and the remaining 60% length of long nephrons whose loops of Henle bend deep in the inner medulla of nephrons are assumed to be water impermeable. To achieve the required permeability in the deeper nephrons ($Lp_{new}^{DL\;IM}$), we calculate at each depth the fraction of descending limbs reaching the last 60% of their length *F*_60%_, and penalize the overall water permeability accordingly: $Lp_{new}^{DL\;IM}=(1-F_{60\%})\times Lp^{DL\;IM}$; i.e., nephrons reaching the last 60% of their length are considered to have a 0 permeability. This scenario leads to high osmolarity gradients, but the osmolarity does not increase in the inner medulla (Fig 10).

The second alternative scenario assumes no external osmoles in the inner medulla, and also leads to a flat osmolarity gradient in the inner medulla.

The third scenario assumes no ammonia production.

Finally, the last scenario assumes that ammonia secretion in the cortical parts of the nephrons is proportional to the rate of delivery, i.e., that it is proportional to the ammonia flow at the exit of the ascending limbs. The constant of proportionality is set at 1.7 based on Wilcox et al. [34] (the equivalent proportionality constant in the initial baseline scenario would be 1.46). This alternative baseline scenario leads to a total cortical production of ammonia of 60 nmole/min, which is higher than in the original baseline scenario (38 nmole/min). Urinary excretion of ammonia was also slightly increased (additional excretion 7 nmole/min).

The parameters having the largest effects on urinary ammonia excretion in each case are presented in Table 3. In the four additional scenarios tested, the parameters and mechanisms that had the greatest effects on urinary excretion were similar to those described above. Alkalinization of interstitial pH was also highly correlated to ammonia excretion (results not shown).

**Table 3:** Parameter analyses based on three alternative baseline scenarios.

Scenarios							
DL partly impermeable		No external osmoles		No production		Cortical Amm	
${\text{Vmax}}_{{\text{NH}}_{4}^{+}}^{\text{AL}}$	++++	${\text{Vmax}}_{{\text{NH}}_{4}^{+}}^{\text{AL}}$	++++	${\text{Vmax}}_{{\text{NH}}_{4}^{+}}^{\text{AL}}$	++++	${\text{P}}_{{\text{NH}}_{3}}^{\text{DL OS}}$	++++
${\text{P}}_{{\text{NH}}_{3}}^{\text{DL OS}}$	++++	${\text{P}}_{{\text{NH}}_{3}}^{\text{DL OS}}$	++++	${\text{P}}_{{\text{NH}}_{3}}^{\text{DL OS}}$	++++	${\text{Vmax}}_{{\text{NH}}_{4}^{+}}^{\text{AL}}$	++++
${\text{P}}_{{\text{NH}}_{4}^{+}}^{\text{AL IS}}$	++	${\text{P}}_{{\text{NH}}_{4}^{+}}^{\text{AL IS}}$	++	${\text{P}}_{{\text{NH}}_{4}^{+}}^{\text{DVR OS}}$	++	${\text{P}}_{{\text{NH}}_{4}^{+}}^{\text{DVR OS}}$	+++
${\text{P}}_{{\text{NH}}_{4}^{+}}^{\text{DVR OS}}$	++	${\text{P}}_{{\text{NH}}_{4}^{+}}^{\text{DVR OS}}$	++	${\text{P}}_{{\text{NH}}_{4}^{+}}^{\text{AL IS}}$	++	${\text{P}}_{{\text{NH}}_{4}^{+}}^{\text{AL IS}}$	+++
		${\text{P}}_{{\text{NH}}_{3}}^{\text{CD IS}}$	+	${\text{P}}_{{\text{NH}}_{3}}^{\text{CD IS}}$	+	${\text{P}}_{{\text{NH}}_{3}}^{\text{CD IS}}$	+
				${\text{P}}_{{\text{NH}}_{3}}^{\text{DL IS}}$	+		
				${\text{P}}_{{\text{NH}}_{3}}^{\text{AL IS}}$	-		
				${\text{P}}_{{\text{NH}}_{4}^{+}}^{\text{DL OS}}$	-		

‘DL partly impermeable’: in this scenario, some sub-segments of the descending limbs are assumed to be water impermeable (the outer-stripe of superficial nephrons, the inner medullary segment of juxtamedullary nephrons whose loop of Henle bends within the first millimeter of the inner medulla, and the last 60% of inner medullary segment of juxtamedullary nephron which go deeper in the inner medulla). ‘no external osmoles’: in this scenario, the external osmoles concentrations in the inner medulla are set to 0. ‘No production’: ammonia production is set to 0. ‘Cortical Amm’: in this scenario, ammonia delivered to the collecting ducts is proportional to ammonia flow at the exit of the ascending limbs. The number of ‘+’ in ‘-’ corresponds to the percentage change of the rate of ammonia excretion from the corresponding baseline scenario: ‘-’ for -20% to -10%; ‘+’ for 10% to 20%; ‘++’ for 20% to 30%; ‘+++’ for 30% to 40%; ‘++++’ for > 40%.

## Discussion

We present here the first mathematical model of ammonia flows in the renal medulla. The model was designed to study the role of each tubular segment and to identify the parameters controlling the fractional excretion of ammonia in normal hydropenia. Simplifications were made (pH and potassium concentrations fixed for each simulation, tube lining modeled as a simple membrane), yet, despite these simplifications, the model showed good agreement with experimental and theoretical data. The predicted ammonia concentrations and fractional deliveries in the baseline scenario are within the range of micropuncture measurements (Fig 3, [10, 11, 18–21]) and reproduce results from microperfusion experiments [21]. Our results emphasize that NH_3_ secretion in the outer medullary collecting ducts is of prime importance for ammonia excretion. Increased ammonia excretion depends on those parameters that increase ${\text{NH}}_{4}^{+}$ reabsorption from the thick ascending limbs and NH_3_ secretion into the outer medullary descending limbs (recycling effect) and collecting ducts. In particular, alkalinization of the interstitium in the outer medulla and acidification of the inner medullary collecting ducts lead to this effect. Several membrane parameters also affect urinary ammonia levels: e.g. the rate of active ${\text{NH}}_{4}^{+}$ transport in the thick ascending limbs, and NH_3_ permeability of the outer stripe descending limbs. These results hold for the various baseline scenarios tested.

### Comparison with published computational models of isolated tubules

In this medullary model, we treated the epithelial walls of the nephrons as simple membranes rather than as full epithelia. This simplification allowed us to simulate all the medullary segments (including the vasa recta) and the interstitium, while maintaining a manageable number of model variables, thereby limiting the uncertainty related to unknown parameters. This approach, however, has its own shortcomings. In particular, it does not allow one to couple sodium, potassium, and ammonium transport, which could limit the validity of our results, especially under conditions affecting the medullary osmotic gradients. To assess some of the limitations, one may compare our results with those of two previous models that explicitly treated luminal pH (but not interstitial pH) of the descending limbs (but not of the other medullary tubes and vessels). The first model, developed by Mejia and colleagues [35], used a membrane approach to investigate alkalinization of the descending limbs; they concluded that NH_3_ entry plays the dominant role in LDL alkalinization. More recently Weinstein published a detailed epithelial model of the proximal tubule and loop of Henle [36]. Whereas the work by Mejia et al. suggests an increase in total ammonia flow along the descending limbs, the work by Weinstein shows an increase of ${\text{NH}}_{4}^{+}$ flow in the outer descending limbs and a decrease in the inner part, which is similar to our findings. In the inner medullary ascending limbs, Weinstein’s model predicts that ${\text{NH}}_{4}^{+}$ is reabsorbed [36], whereas our model predicts a slight secretion; this difference can be explained by the differences in ammonia concentrations in the interstitium / bath. In the Weinstein model, the fixed ammonia concentration assumed in the inner medullary interstitium favored reabsorption, which was not our case. Profiles of NH_3_ were not reported in their published results and thus cannot be compared with ours. In the outer medullary ascending limbs, experimental results [21] recognize ${\text{NH}}_{4}^{+}$ uptake as an important factor for urinary excretion and delivery to the outer medullary collecting ducts. Our model reproduces this feature. In the inner medullary collecting ducts, the epithelial model of Weinstein suggested that ammonia flow is relatively constant. We found a similar result in our model.

### Limitations related to the assumption of a fixed pH gradient

Since our model’s predicted concentrations and flows conform well to available experimental measurements of renal medullary ammonia handling, we feel that its predictions concerning the control of urinary ammonia excretion have merit. Nonetheless, future models of medullary ammonia handling would do well to go further, at the expense of increased model complexity. In particular, the most important improvement would be to treat interstitial and tubular pH as an explicit model variable. At a minimum, this would require the addition of bicarbonate and CO_2_ as explicit solutes. One previous model [35] did this successfully, though only for the LDL and only for the luminal pH (i.e., interstitial pH was fixed, as in our model). Recent results (e.g., the role of sulfatides in renal ammonium handling [37]) suggest that to do it properly, one would need to treat both intraluminal and interstitial changes of pH as a function of varying concentrations and transmembrane fluxes. Given the considerable number of additional parameters involved, many of which have not been experimentally measured, we decided not to build in the added complexity in this first model.

### Transepithelial ammonia fluxes in outer medulla versus inner medulla

Our model predicts that total ammonia secretion in the collecting ducts occurs in the outer medulla but not (or little) in the inner medulla. These results are in line with the results obtained using a detailed cellular model of the collecting duct [38], yet, it is usually considered that the (terminal) inner medullary CD is an important site for secretion / excretion. This hypothesis notably comes from the observation of a positive NH_3_ concentration gradient between the interstitium and the collecting ducts [27]. However, in our simulations, the passive gradient for NH_3_ was reversed in the IM (absorptive fluxes), notably due to a difference in our pH hypothesis. Indeed, controversy exists regarding the value of interstitial pH in the papilla. Kersting and colleagues [39, 40] reported a pH of ∼ 6.44–6.71 in vasa recta (a proxy for interstitial pH) much lower than the value reported by Dubose et al. (pH 7.28 in [27]). Since the pKa for ammonia is 9.05, a more acidic interstitium results in a lower NH_3_ concentration, and diminishes (or reverses) the transepithelial concentration gradient for diffusion into the collecting ducts. For the baseline simulations, we chose a scenario between Kersting and Dubose (interstitial pH in papilla 6.9), but we explored a range of values in our parameter studies. A second difference between the model predictions and the Dubose study concerns the total concentration of ammonia in the collecting ducts. Our predictions are compatible with the literature, but higher than those of Dubose’s study. As a result, we obtain a (small) NH_3_ transepithelial gradient favoring reabsorption from collecting ducts. In any case, the model predicted insignificant NH_3_ transmural fluxes in the IMCD, because the surface area available for exchange (number of tubes) and NH_3_ permeability are low in this region (∼10 fold lower than in OMCD [22, 41]). As a consequence, high NH_3_ secretion appears unlikely in the terminal CD. Another observation in favor of the role of the IMCD for ammonia excretion comes from the studies by Wall and co-workers [25]. Wall et al. showed that ${\text{NH}}_{4}^{+}$ is actively transported by Na-K-ATPase. Even when our assumptions (rate of transport, apparent affinity) are similar to those of Wall and Koger and lead to the same order of magnitude of ${\text{NH}}_{4}^{+}$ fluxes, ${\text{NH}}_{4}^{+}$ secretion in IMCD was not significant. Our model therefore suggests that the terminal inner medulla is not a major site of ammonia excretion.

### How to increase ammonia excretion

In this study, several medullary parameter modifications led to an increase in the rate of ammonia excretion: an alkalinization of OM interstitium, an increase in the rate of ${\text{NH}}_{4}^{+}$ active transport in the MTAL, and an increase in NH_3_ permeability in DL OS. It must be noted that cortical segments were not included in the model, though the ammonia production of the distal tubules reported in the literature is included as boundary conditions for CD inflow (see Eqs 21 and 22). Concerning the role of distal nephron segments within the cortex, the current model explores only scenarios in which these segments secrete ammonia, as observed in experimental studies. However, the literature on cortical ammonia handling is sparse, and cortical ammonia reabsorption cannot be excluded. It is therefore possible that the cortical segments play a more complex role in ammonia metabolism than is currently thought and modelled here, notably in the cortical collecting ducts. Further experimental and theoretical studies are required to elucidate this point. The medullary parameters impacted ammonia excretion via a commonly invoked mechanism: they increased ammonia reabsorption in the thick ascending limbs, which promoted secretion into the collecting ducts and recycling into the descending limbs. The model helps to better understand how ammonia recycling in the loops of Henle promotes urinary ammonia excretion. Ammonia recycling works because it increases ammonia delivery to the MTAL and not because it favors inner medullary accumulation and IMCD secretion. For this process, short nephrons appear particularly important since the model predicts that 72% of ammonia reabsorption in the MTAL is mediated by superficial nephrons. This is because short nephrons are more numerous than deep nephrons and because ammonia concentrations at the entry of MTAL of these nephrons are higher. The role of ammonia recycling in the short loop of Henle was also noted by Weinstein in a recent paper [36], which developed a detailed epithelial model of the loop of Henle. The challenge now is to determine which of these predicted factors are involved in urinary excretion in vivo. In the model, NH_3_ transmural fluxes, which depend on pH, appear to be quantitatively important. Experimental studies showed that ammonia excretion was correlated with urine pH and with the gradient of NH_3_ concentrations between the interstitium and the collecting ducts (gradient which depends on the pH environment) [30, 42, 43]. Therefore, a physiological regulation based on pH seems plausible. Regarding the rate of active transport in the MTAL, Attmane-Elakeb and co-authors [44] have shown that ${\text{Na}}^{+}{\text{K}}^{+}({\text{NH}}_{4}^{+})-2{\text{Cl}}^{-}$) is upregulated in acidosis. This suggests that an increase in ${\text{NH}}_{4}^{+}$ uptake in MTAL is a plausible candidate to control urinary excretion. It must be noted, however, that our approach does not allow the exploration of the role of specific transporters. To obtain such results, an epithelial approach, such as Weinstein’s study of the catalytic role of ammonia in sodium reabsorption in the thick ascending limbs [45], is required. However, such an approach requires the specification of many more parameters, and has not yet been done in a model of the whole medulla. As a first approximation, our model is sufficient to explore the role of each tubular segment. Further studies including detailed epithelial transport and explicit treatment of the 3-dimensional structure of the medulla could help to refine our results. The last parameter having a large impact on urinary excretion is the NH_3_ permeability in the DL, since it favors NH_3_ recycling. The transport mechanisms in the descending limbs are not well characterized, so it is difficult to assess the plausibility. In the model, increasing NH_3_ permeability in the CD does not increase ammonia urinary excretion, but inhibiting this permeability limits it, as reported in mice with Rhbg and Rhcg knock-outs [23].

### Competition with potassium

In the model, low potassium concentrations in the medulla lead to a relatively mild increase in urinary ammonia excretion (+18%) through an increase in ammonia reabsorption in MTAL. ${\text{NH}}_{4}^{+}$ transmural fluxes are also increased in CD, but their contribution to urinary flow of ammonia remains marginal. It is difficult to compare these values with the literature, since pathologic changes in plasma potassium levels are known to affect not only potassium concentration gradients, but also the reabsorption of water and sodium, renal ammonia production and the expression of ammonia transporters [19, 27, 46–50]. Hence, our results only represent the isolated effect of potassium, and not a physiological scenario.

In summary, we developed the first model of medullary ammonia transport in the rat to study the role of each tubular segment. Our results suggest that the principal mechanisms controlling ammonia excretion are located in the outer medulla and favor ammonia recycling in the loops of Henle.

## Methods

### Physiological basis for the model

The assumptions regarding renal ammonia handling are based on the following understanding. Between 60% and 80% of renal ammoniagenesis occurs in the cells of the proximal tubules, and the rest is produced in other tubular segments [15]. In the thin descending limbs, ammonia is secreted across the epithelium into the lumen by passive diffusion, but the species transported (NH_3_ or ${\text{NH}}_{4}^{+}$) is unknown [18, 51]. In the thick ascending loops of Henle, ${\text{NH}}_{4}^{+}$ is transported from the lumen into the interstitium; 65% of the transport is mediated by (secondary) active transport through the apical Na^+^-K^+^-2Cl^−^ cotransporter NKCC2 (competition with potassium) and basolateral Na^+^-H^+^ exchanger NHE4 (competition with hydrogen) [21, 52, 53]. As a result, the amount of ammonia entering the distal tubules is only 20–30% of the amount entering the descending limbs, despite significant production along the loops of Henle (concentration around 1mM in early distal tubule [11, 12]). Superficial distal tubules secrete only a small amount of ammonia under normal conditions [11, 34]. A large fraction (between 40% and 80%) of excreted urinary ammonia is from secretion into the collecting ducts (CD), notably via Rh C glycoprotein (Rhcg), a specific NH_3_ transporter [23]. Rhcg is mainly expressed in the cortex and outer stripe, but is also present in the inner stripe and inner medullary collecting ducts (IMCD) [54, 55]. In the IMCD, ${\text{NH}}_{4}^{+}$ may also be transported via basolateral Na^+^-K^+^-ATPase (competition with potassium) [19, 25].

From the mechanisms of transport described above, ${\text{NH}}_{4}^{+}$ and NH_3_ medullary transport and resulting urinary ammonia excretion depend on at least three factors: first, the expression of NH_3_ and ${\text{NH}}_{4}^{+}$ carriers (potassium channels, NKCC2, Rhcg, Na^+^-K^+^-ATPase); second, the cortico-medullary pH gradients along the tubule lumens and within the interstitium; and finally, the luminal and interstitial potassium concentrations. The first factor (expression of transporters) may be the best understood due to the numerous experimental techniques developed; in particular, isolated microperfusion studies and knockout models. On the other hand, the renal medullary microenvironment remains inaccessible, and the roles of interstitial pH and potassium concentrations have not been fully elucidated. Despite the very low concentration of NH_3_ relative to ${\text{NH}}_{4}^{+}$ (pKa 9.05), movements of NH_3_ may be quantitatively important due to the differences between NH_3_ and ${\text{NH}}_{4}^{+}$ permeabilities. The general changes in pH along the tubular segments have been described in the cortical and papillary regions [18, 30, 40]. However, pH gradients within the medulla remain uncertain, and controversy remains regarding the pH at the bottom of the inner medulla [39]. Our modeling analysis is thus focused on gauging the role of the pH and potassium concentrations in ammonia handling.

### Mathematical model

#### Overview

The model illustrated in Fig 1 represents the transport of water and solutes (NaCl, urea, NH_3_, ${\text{NH}}_{4}^{+}$, and an unspecified non-reabsorbable solute (NRS) which includes the effect of KCl in the collecting ducts) in an idealized rat renal medulla. This model is an adaptation of the steady state 2-D model of Hervy and Thomas [17], which was developed to investigate the influence of glycolytic lactate production on the inner medullary osmotic gradient. The aim of the current model, however, is not to investigate the urine concentrating mechanism, but rather to focus on medullary ammonia transport. Consequently, the description of the concentrating mechanism is phenomenological. In particular, the inner medullary osmolality gradient is generated artificially by introduction of virtual external osmoles (see [17, 56, 57]), and the thin descending limbs are assumed to be water permeable along their whole length. The impact of these hypotheses is explored at the end of the results section, where we present the results of a partial sensitivity analysis based on alternative baseline scenarios.

The model equations describe the variations in volume and solutes flows resulting from diffusion gradients and active transport. Differences of electrical potential, pH, and potassium concentration gradients are imposed at each depth. These gradients directly affect NH_3_ and ${\text{NH}}_{4}^{+}$ transmural fluxes. It must be noted that sodium transport is considered here to be independent of potassium gradients, and therefore our model may not be suitable to study the impact of large variations in potassium concentrations (see discussion). Similarly, the independence between medullary pH and NH_3_ and ${\text{NH}}_{4}^{+}$ movements is a simplification, since medullary pH is collectively determined by bicarbonate, CO_2_, carbonic anhydrase concentrations, ammonia, and titrable acids. Model outputs of each simulation scenario give the concentration and flow profiles along each medullary structure. Most of the parameter values were taken from the rat literature. To identify the parameters associated with a change in urinary ammonia excretion, a partial sensitivity analysis was performed; starting from our baseline (control) scenario, each parameter value (e.g., NH_3_ permeability in the outer stripe collecting duct) was perturbed and the changes in renal ammonia transport were analyzed. The model is coded in C (gcc compiler) and uses the gnu scientific library (gsl) for numerical calculations.

#### Model topology

In the model, the renal medulla is composed of nephrons, collecting ducts, and vasa recta, all of which are bathed in a common interstitium (see Fig 1). A nephron is modeled as a loop of Henle and comprises descending and ascending segments. The model does not include cortical elements such as distal tubules; instead, as in many such models of the renal medulla, inflow to the outer medullary collecting ducts is calculated from the flow leaving the ascending limbs based on mass balance and reasonable assumptions of distal tubule processing. Two types of nephron are distinguished: short nephrons, whose loops of Henle turn at the outer-inner medullary border, and long nephrons, which go deeper into the inner medulla. The vasa recta are also composed of descending and ascending segments. The ascending vasa recta are lumped with the interstitium, i.e., concentrations in the ascending vasa recta and in the interstitium are supposed equal. As is usual in medullary models to date, the tube lining is assumed to be a simple membrane rather than a cellular epithelium with apical and basolateral properties.

#### Number of tubes

The numbers of nephrons, collecting ducts, and vasa recta vary with depth within the medulla, reflecting data from studies of rat kidney anatomy (Table 4). As in Hervy and Thomas [17] and in many models by other authors, we did not explicitly model each of the 16,000 short nephrons or all of the collecting ducts. Instead, we represent each type of structure by a single lumped tubular structure. To take into account the number of tubes at a given depth, the circumference of each lumped structure reflects the total number of tubes at that depth. Explicitly, the circumference is given by 2*πr* ^*t*^ *N* ^*t*^(*x*) where *r* ^*t*^ is the radius of the tube and *N* ^*t*^(*x*) the number of tubes at depth *x*. The circumference, which is numerically equivalent to the membrane area per unit of tube length *A* ^*t*^(*x*), defines the surface area available for exchanging volume and solutes between the tube and the interstitium. In the inner medulla, the numbers of nephrons and vasa recta decrease with depth towards the papillary tip, and therefore the flows in the lumped structures must also decrease. To model this, we include virtual shunts: flows of water and solutes in the descending limbs of the loops of Henle and descending vasa recta are shunted directly to their ascending counterparts in proportion to the number of tubes that return at each depth (Eq 1 and for instance Eqs 2 and 3, Fig 1). Thus the shunt flux is:

$$F_{shunt}^{t}\left(x\right)=\frac{F^{t}\left(x\right)}{N^{t}\left(x\right)}\frac{dN^{t}\left(x\right)}{dx}$$ (1)

where *F* ^*t*^(*x*) represents the flow of water or solute in tube *t* at depth *x*.

**Table 4:** Number of tubes at each depth *x*.

	OM OS	OM IS	IM
LDL and LAL	$2N_{0}^{CD}$	$2N_{0}^{CD}$	$N_{0}^{LDL}e^{-1.213(x-x_{OM/IM})}$
SDL and SAL	$4N_{0}^{CD}$	$4N_{0}^{CD}$	-
DVR	0.6$\left(N_{0}^{LDL}+N_{0}^{SDL}\right)$	0.6$\left(N_{0}^{LDL}+N_{0}^{SDL}\right)-$0.6$N_{0}^{SDL}\frac{\left(x-x_{OS/IS}\right)}{\left(x_{OM/IM}-x_{OS/IS}\right)}$	0.6*N* ^*LDL*^(*x*)
CD	$N_{0}^{CD}$	$N_{0}^{CD}$	$N_{0}^{CD}e^{-1.04(x-x_{OM/IM})}$

The numbers of tubes at each depth *x* are defined relative to the number of collecting ducts, $N_{0}^{CD}\;=\;4000$ (total number of tubes: 24000 nephrons and 48000 vasa recta) [58]. In the outer medulla, the ratio of nephrons to collecting ducts is assumed to be 6:1. The ratio of short to long nephrons is assumed to be 2:1. The total number of nephrons and collecting ducts are assumed to decrease exponentially in the inner medulla (decay *λ* ^*loop*^ = -1.213 and *λ* ^*CD*^ = -1.04). The ratio of the number of descending vasa recta to nephrons is taken to be 0.6:1 at x = 0 [59]. The position of the OS-IS junction is taken as 0.7 mm, the OM/IM junction as 2 mm, the upper/lower inner medulla junction at 3.3 mm, and the total length of the medulla as 6mm [58, 60].

### Equations for transmural transport of water and solutes

#### Volume flows

The following equations describe the variations ($\frac{dF_{v}^{t}}{dx}$) in volume flows of water with depth in tube *t* at steady state [61]. Transmural fluxes of water from the tubular lumen to the interstitium, *J*_*v*_, are driven by the effective osmotic pressure difference:

$$\begin{array}{ccc} \frac{dF_{v}^{DL}\left(x\right)}{dx} & = & -J_{v}^{DL}\left(x\right)-F_{shunt,v}^{DL}\left(x\right) \end{array}$$ (2)

$$\begin{array}{ccc} \frac{dF_{v}^{AL}\left(x\right)}{dx} & = & -J_{v}^{AL}\left(x\right)+F_{shunt,v}^{DL}\left(x\right) \end{array}$$ (3)

$$\begin{array}{ccc} \frac{dF_{v}^{DVR}\left(x\right)}{dx} & = & -J_{v}^{DVR}\left(x\right)-F_{shunt,v}^{DVR}\left(x\right) \end{array}$$ (4)

$$\begin{array}{ccc} \frac{dF_{v}^{CD}\left(x\right)}{dx} & = & -J_{v}^{CD}\left(x\right) \end{array}$$ (5)

$$\begin{array}{ccc} J_{v}^{t}\left(x\right) & = & A^{t}\;Lp^{t}R\;T\;[\sum_{s}\sigma_{s}^{t}\gamma_{s}(c_{s}^{Int}\left(x\right)-c_{s}^{t}\left(x\right))-E\left(x\right)] \end{array}$$ (6)

where *F*_*shunt*_ is defined in Eq (1), *A* ^*t*^ is membrane area per unit of length of tubular segment *t*, *Lp* ^*t*^ the water conductivity, *R* is the ideal gas constant, *T* the absolute temperature, $\sigma_{s}^{t}$ is the reflexion coefficient for solute *s*, *γ*_*s*_ the activity coefficient for solute *s* (1.0 for urea, non reabsorbable solute (NRS) and NH_3_, and 1.82 for NaCl, ${\text{NH}}_{4}^{+}$), *E*(*x*) the concentration of external osmoles, $c_{s}^{Int}(x)$ and $c_{s}^{t}(x)$ are the concentrations of solute *s* in the interstitium and tubular segment *t*. DL (AL) labels the short and long descending (ascending) limbs, respectively.

As in Thomas and Wexler [57], external osmolytes *E*(*x*) are introduced in the inner medulla to increase inner medullary osmolality, thus drawing water from descending limbs and collecting ducts, concentrating their solutes and leading to solute accumulation in the papilla by countercurrent exchange. We emphasize that since the medullary osmolality gradient is important for renal ammonia handling but the true mechanism responsible for building an inner medullary osmolality gradient is unknown, we used this surrogate mechanism, which has precedents in the literature. The value of *E*(*x*) is fixed during a given simulation.

#### Solute flows

Changes in solute flows ($F_{s}^{t}$) are due to transmural movement or tubular production ($Prod_{s}^{t}(x)$). Transmural movement (i.e. flux) of solutes can be driven by diffusion (*J*_*diff*_), convection (*J*_*conv*_), or by active transport (*J*_*active*_). Fluxes are defined as positive for the lumen-to-interstitium direction, i.e., positive for (re-)absorption, and negative for tubular secretion. Differential equations governing fluxes of solutes from the tubular lumen to the interstitium in the loops of Henle and vasa recta are as follows:

$$\begin{array}{ccc} \frac{dF_{s}^{DL}\left(x\right)}{dx} & = & -J_{conv,s}^{DL}\left(x\right)-J_{\text{diff},s}^{DL}\left(x\right)-F_{shunt,s}^{DL}\left(x\right)+Prod_{s}^{DL}\left(x\right)\\ \; \end{array}$$ (7)

$$\begin{array}{ccc} \frac{dF_{s}^{AL}\left(x\right)}{dx} & = & -J_{conv,s}^{AL}\left(x\right)-J_{\text{diff},s}^{AL}\left(x\right)-J_{active,s}^{AL}\left(x\right)+F_{shunt,s}^{DL}\left(x\right)+Prod_{s}^{AL}\left(x\right) \end{array}$$ (8)

$$\begin{array}{ccc} \frac{dF_{s}^{DVR}\left(x\right)}{dx} & = & -J_{conv,s}^{DVR}\left(x\right)-J_{\text{diff},s}^{DVR}\left(x\right)-F_{shunt,s}^{DVR}\left(x\right)+Prod_{s}^{DVR}\left(x\right) \end{array}$$ (9)

where *s* ∈ {NaCl, urea, non reabsorbable solute, NH_3_, ${\text{NH}}_{4}^{+}\}$, and the superscripts DL, AL, and DVR represent the descending and ascending limbs of the loops of Henle, and descending vasa recta, respectively. The production term $Prod_{s}^{t}(x)$ is zero for all solutes except ammonia (see Table 5).

**Table 5:** Rates of total ammonia production in the various tubular segments (pmol.min^-1^.mm^-1^ tube) [15].

	DL	AL	CD
OS	2.3	3.5	0.8
IS	0.3	3.5	0.8
UIM	0.3	0	0.8
LIM	0.3	0	0.8

The convection term (aka solvent drag) is given by:

$$J_{conv,s}^{t}\left(x\right)=J_{v}^{t}\left(x\right)\left(1-\sigma_{s}^{t}\right)\frac{c_{s}^{t}\left(x\right)+c_{s}^{Int}\left(x\right)}{2}$$ (10)

The diffusion of non-ionic species is given by:

$$J_{\text{diff},s}^{t}\left(x\right)=A^{t}\;P_{s}^{t}\;(c_{s}^{t}\left(x\right)-c_{s}^{Int}\left(x\right))$$ (11)

where $P_{s}^{t}$ is the permeability coefficient.

The diffusion of the ionic species (e.g. ${\text{NH}}_{4}^{+}$) is given by the Goldman Hodgkin Katz flux equation:

$$J_{\text{diff},s}^{t}\left(x\right)=A^{t}\;P_{s}^{t}\frac{z_{s}𝓕\Delta V\left(x\right)}{RT}\;(\frac{c_{s}^{t}\left(x\right)-c_{s}^{Int}\left(x\right)e^{\frac{-𝓕\Delta V(x)}{RT}}}{1-e^{\frac{-𝓕\Delta V(x)}{RT}}})$$ (12)

where z_*s*_ is the valence of ion s, ℱ is the Faraday constant, Δ*V* the transmembrane electric potential difference (see Table 6).

**Table 6:** Model parameters.

		r	LpRT	P_*Na*_	P_*u*_	P_*NRS*_	${\text{P}}_{NH_{4}^{+}}$	P_*NH*_3__	*σ*_*Na*_	*σ*_*u*_	*σ*_*NRS*_	$\sigma_{NH_{4}^{+}}$	*σ*_*NH*_3__	Vmax_*Na*_	Km_*Na*_	${\text{Vmax}}_{NH_{4}^{+}}$	${\text{Km}}_{NH_{4}^{+}}$	Km_*K*^+^_	ΔV
LDL	OS	10	67	20	2	0	10	2000	0.9	1	1	0.9	0.9	0	0	0	0	0	0
	IS	10	63	20	2	0	30	2000	1	1	1	1	1	0	0	0	0	0	0
	UIM	10	58	1	12	0	15	500	1	1	1	1	1	0	0	0	0	0	0
	LIM	10	58	0.5	12	0	100	1800	1	1	1	1	1	0	0	0	0	0	
LAL	OS	10	0	2	4.5	0	6	100	1	1	1	1	1	0.201	50	0.0221	2	1	15
	IS	10	0	2	4.5	0	6	100	1	1	1	1	1	0.201	50	0.0221	2	1	15
	UIM	10	0	80	23	0	200	3000	1	1	1	1	1	0	50	0	0	0	0
	LIM	10	0	80	23	0	130	1600	1	1	1	1	1	0	50	0	0	0	0
SDL	OS	11	58	2.3	8.5	0	10	2000	1	1	1	1	1	0	0	0	0	0	0
	IS	11	50	1	8.4	0	30	2000	1	1	1	1	1	0	0	0	0	0	0
SAL	OS	10	0	2	4.5	0	6	100	1	1	1	1	1	0.227	50	0.0221	2	1	15
	IS	10	0	2	4.5	0	6	100	1	1	1	1	1	0.227	50	0.0221	2	1	15
CD	OS	15	10	0	0.5	0	0.5	4000	1	1	1	1	1	0.005	50	0.0225	10	2	-20
	IS	15	5	0	0.5	0	0.1	1000	1	1	1	1	1	0.005	50	0.0006	10	2	-20
	UIM	15	3	0	1	0	0.05	200	1	1	1	1	1	0.005	50	0.0006	10	2	-20
	LIM	15	3	0	70	0	0.01	200	1	1	1	1	1	0.005	50	0.0006	10	2	-20
DVR	OS	9	67	80	360	0	80	400	0.5	0.5	0.5	0.5	0.5	0	0	0	0	0	0
	IS	9	25	80	360	0	80	400	0.5	0.5	0.5	0.5	0.5	0	0	0	0	0	0
	UIM	9	33	80	120	0	75	400	0.5	0.5	0.5	0.5	0.5	0	0	0	0	0	0
	LIM	9	33	80	120	0	75	400	0.5	0.5	0.5	0.5	0.5	0	0	0	0	0	0

r: tube radius (*μ*m), Lp: hydraulic permeability, R: gas constant, T temperature (LpRT in 10^-6^mm.s^-1^.mosM^-1^), P permeability coefficient (10^-5^cm.s^-1^), *σ* reflexion coefficient, Vmax in nmole.mm^-2^.min^-1^, Km in mM, ΔV: transmembrane electrical potential in mV, Na: sodium, u: urea, NRS: non reabsorbable solute (see text for reference).

Active transport is modeled using saturable irreversible Michaelis-Menten kinetics:

$$J_{active,s}^{t}\left(x\right)=A^{t}\frac{Vmax_{s}^{t}\;c_{s}^{t}\left(x\right)}{Km_{s}^{t}+c_{s}\left(x\right)}$$ (13)

where $Vmax_{s}^{t}$ is the rate of active transport at saturated concentration of solute *s* and ${\text{Km}}_{s}^{t}$ is the concentration of solute *s* at half-maximal transport rate.

Flows along the CD are given by:

$$\begin{array}{ccc} \frac{dF_{NaCl}^{CD}\left(x\right)}{dx} & = & -J_{\text{diff},NaCl}^{CD}\left(x\right)-J_{conv,NaCl}^{CD}-J_{active,NaCl}^{CD}\left(x\right) \end{array}$$ (14)

$$\begin{array}{ccc} \frac{dF_{NH_{4}^{+}}^{CD}\left(x\right)}{dx} & = & -J_{\text{diff},NH_{4}^{+}}^{CD}\left(x\right)-J_{conv,NH_{4}^{+}}^{CD}+J_{active,NH_{4}^{+}}^{CD}\left(x\right)+Prod_{NH_{4}^{+}}^{CD}\left(x\right) \end{array}$$ (15)

$$\frac{dF_{s}^{CD}(x)}{dx}=-J_{diff,s}^{CD}(x)-J_{conv,s}^{CD}\;\;s\in \{\text{urea},{\text{NH}}_{\text{3}},\text{non}\;\text{reabsorbable}\;\text{solute}\}$$ (16)

For $J_{active,NH_{4}^{+}}^{CD}$, the model uses interstitial ${\text{NH}}_{4}^{+}$ concentrations (${\text{NH}}_{4}^{+}$ is transported from the interstitium to the lumen). The parameter values depend on the region (see Table 6).

#### Mass balance

As shown by Stephenson [61, 62], steady state mass balance for flows in a system of rigid counter-flowing tubes with a single exit at the bottom (here, the collection ducts), requires that at each depth, *x*, and for each solute, *i*, the sum of the flows in all tubes of any given solute or of volume, (taking flow to be positive towards the papilla and negative away from the papilla) must equal the outflow from the terminal CD plus the total amount of solute *i* synthesized from *x* to the papillary tip *x* = *L* (rat medullary length taken as *L* = 6 mm), as given in the following equation:

$$\sum_{t}F_{i}^{t}\left(x\right)=F_{i}^{CD}\left(L\right)+\sum_{t}\int_{L}^{x}\;Prod_{i}^{t}\left(u\right)du.$$ (17)

This mass balance condition was used here as the criterion for convergence of the numerical iteration scheme described above.

### Assumptions specific to ammonia handling

The model calculates the changes in total ammonia ($\text{tAmm}={\text{NH}}_{3}+{\text{NH}}_{4}^{+}$) resulting from NH_3_ and ${\text{NH}}_{4}^{+}$ transmural transport and total ammonia production. At each depth, the concentrations of [NH_3_] and [${\text{NH}}_{4}^{+}$] are calculated from the concentration of total ammonia [tAmm] and the tubular pH, using the Henderson-Hasselbalch equation

$$\begin{array}{ccc} \left[NH_{3}\right] & = & \frac{10^{pH-pKa}}{1+10^{pH-pKa}}\left[tAmm\right] \end{array}$$ (18)

$$\begin{array}{ccc} \left[NH_{4}^{+}\right] & = & \left[tAmm]-[NH_{3}\right] \end{array}$$ (19)

where pKa is the dissociation constant of ammonia (pKa = 9.05). The pH values are specified at each depth for each tubular segment and remain fixed during a given simulation (see Table 7). Transmural fluxes are calculated for each species (*J*_*NH*_3__ and $J_{NH_{4}^{+}}$) and added to determine the transmural flux of total ammonia ($J_{tAmm}=J_{NH_{3}}+J_{NH_{4}^{+}}$). ${\text{NH}}_{4}^{+}$ is assumed to be transported by active transport along thick ascending limbs (outer medulla) and the collecting ducts [21, 25]. For the thick ascending limbs, the rate-limiting step is assumed to be the uptake by NKCC2 on the luminal side (Km similar to the Km value of NKCC2 for ${\text{NH}}_{4}^{+}$ as measured in rabbit [63]). For the collecting ducts, the Km is associated with Na^+^-K^+^-ATPase. Competition between ${\text{NH}}_{4}^{+}$ and *K* ^+^ in the thick ascending limbs and collecting ducts is included by modifying the apparent affinity of ${\text{NH}}_{4}^{+}$ for its transporter (see Table 6):

$$Km_{app}=Km_{NH_{4}^{+}}\left(1+\frac{\left[K^{+}\right]}{Km_{K^{+}}}\right)$$ (20)

**Table 7:** pH gradients assumed in the model.

	Top OM	border OM/IM	borderUIM/LIM	Bottom IM
LDL	6.85			7.39
LAL	6.6			7.39
SDL	6.85	7.03		
SAL	6.77	7.03		
CD	6.6		6.15	5.99
DVR	7.34		7.20	6.90
Int/AVR	7.35		7.10	6.90

pH assumptions based on [11, 18, 30, 40, 64, 65]. pH values are defined at the entry and at the exit of each tube, and in some cases at the junction between the outer and inner medulla or upper and lower inner medulla. Linear interpolation is used to set pH elsewhere.

Potassium concentrations [*K* ^+^] at each depth are specified at the beginning of each simulation (see Table 8).

**Table 8:** Potassium gradients (mM) assumed in the model.

	Top OM	border OM/IM	Bottom IM
MTAL	2.8	26	
Interstitium	4.4		34

Potassium assumptions based on [13, 14, 66, 67]. Potassium concentrations are defined at the entry and exit of the medullary thick ascending limbs (MTAL) and interstitium, and elsewhere linearly interpolated. At the entry of the collecting ducts, potassium concentration is initialized at 20mM and is included in the calculation of osmotic pressure (potassium flow is assumed to be constant in this tube, so its concentration increases as volume flow decreases along the CD) as in [17].

### Numerical scheme

The numerical scheme was developed by Stephenson [62] and is similar to the scheme used in Hervy and Thomas [17]. The medullary length is discretized into 300 slices. In the first step, the interstitial concentrations at each depth are fixed, and tubular flows are calculated in the direction of flow. To calculate the flow from one position to the next, we combined a mid-point discretisation scheme with a Newton-Ralphson solver. Once the tubular flows have been calculated at each depth, the concentrations in the interstitium are updated using a criterion based on mass balance (Eq 17). These two steps are repeated until the difference between the left and right hand side of Eq 17 is less than 10^−10^.

### Baseline scenarios

#### Inputs and Boundary conditions

Inflows at the entry of short and long nephrons, and vasa recta are summarized in Table 9. In the baseline scenario, we assume that the total ammonia concentration [tAmm] at the entry to the descending limbs is 1.7 mM [11]. Plasma ammonia concentration at the entry to the descending vasa recta is assumed to be similar to plasma ammonia levels and is set to 0.1 mM [10]. Ammonia is also produced in the medulla. The rates of production for each tubular segment $Prod_{tAmm}^{t}$ are taken from reference [15] (see Table 5). The flow of ammonia at the entry to collecting ducts $F_{tAmm}^{CD}(0)$ is assumed to equal the sum of the flows leaving the nephrons plus distal production ($Prod_{tAmm}^{DT}$):

$$F_{tAmm}^{CD}\left(0\right)=F_{tAmm}^{SAL}\left(0\right)+F_{tAmm}^{LAL}\left(0\right)+Prod_{tAmm}^{DT}.$$ (21)

**Table 9:** Inflow to the entry to the tubes.

	Fv	[Sodium]	[Urea]	[NRS]	[Total Ammonia]	Osmolality
DL	10	137	10	1	1.7	264
DVR	11	139.46	5	5	0.1	264

Initial concentrations and volume flows F_*v*_ at the entry to the short and long descending limbs (DL) and vasa recta. Volume flow in nL/min, concentration in mM, osmolality in mosm/Kg H_2_O. Sodium concentration is calculated to ensure that osmolality equals 264 at the entry to the descending tubules (activity coefficient 1.82) [17].

The rate of production per distal tubule $Prod_{tAmm}^{DT}$ is set at 1.6 pmol/min per tube as reported [11], which leads to a total distal production given by:

$$Prod_{tAmm}^{DT}=1.6\times (N^{SAL}\left(0\right)+N^{LAL}\left(0\right))$$ (22)

where *N* ^*SAL*^(0) and *N* ^*LAL*^(0) are the numbers of short and long ascending limbs at *x* = 0. The volume inflow into short and long nephrons is taken to be 10 nl/min per tube tube (i.e., as in many earlier studies, this assumes single-nephron-glomerular-filtration rate of 30 nl/min and reabsorption of 2/3 of volume flow along the proximal tubules). Plasma inflow entering each DVR was taken to be 11 nl/min. The external osmoles in the inner medulla are set at 75 mM in the baseline case.

#### Transport parameters

Except for ammonia, essentially all transport parameters were taken from Hervy and Thomas ([17], Table 6). For NH_3_ and ${\text{NH}}_{4}^{+}$, permeability parameters in the loops of Henle and collecting ducts were based on [21, 25, 41, 63, 68–73]. Values for NH_3_ and ${\text{NH}}_{4}^{+}$ permeabilities in the DVR are unknown, so we used permeabilities similar to the values for urea and sodium, respectively. Vmax in AL was set so that ammonia at the exit of the ascending limbs represents ∼20% of ammonia delivery at the entry of the DL; in the collecting ducts, Vmax was taken from [25].
